# Advances in multi-omics for esophageal squamous cell carcinoma: diagnostic, prognostic, and therapeutic perspectives

**DOI:** 10.1093/procel/pwag005

**Published:** 2026-02-04

**Authors:** Dengyun Zhao, Xinyu He, Yaping Guo, Huifang Wei, Zigang Dong, Kangdong Liu

**Affiliations:** Medical Research Center, Chest Hospital of Zhengzhou University, Zhengzhou 450000, China; Department of Pathophysiology, School of Basic Medical Sciences, Zhengzhou University, Zhengzhou 450000, China; China-US (Henan) Hormel Cancer Institute, Zhengzhou 450000, China; Tianjian Laboratory of Advanced Biomedical Sciences, Zhengzhou University, Zhengzhou 450000, China; Department of Pathophysiology, School of Basic Medical Sciences, Zhengzhou University, Zhengzhou 450000, China; China-US (Henan) Hormel Cancer Institute, Zhengzhou 450000, China; Department of Pathophysiology, School of Basic Medical Sciences, Zhengzhou University, Zhengzhou 450000, China; The Collaborative Innovation Center of Henan Province for Cancer Chemoprevention, Zhengzhou 450000, China; State Key Laboratory of Esophageal Cancer Prevention and Treatment, Zhengzhou University, Zhengzhou 450000, China; Department of Pathophysiology, School of Basic Medical Sciences, Zhengzhou University, Zhengzhou 450000, China; China-US (Henan) Hormel Cancer Institute, Zhengzhou 450000, China; Department of Pathophysiology, School of Basic Medical Sciences, Zhengzhou University, Zhengzhou 450000, China; China-US (Henan) Hormel Cancer Institute, Zhengzhou 450000, China; Tianjian Laboratory of Advanced Biomedical Sciences, Zhengzhou University, Zhengzhou 450000, China; The Collaborative Innovation Center of Henan Province for Cancer Chemoprevention, Zhengzhou 450000, China; State Key Laboratory of Esophageal Cancer Prevention and Treatment, Zhengzhou University, Zhengzhou 450000, China; Provincial Cooperative Innovation Center for Cancer Chemoprevention, Zhengzhou University, Zhengzhou 450000, China; Department of Pathophysiology, School of Basic Medical Sciences, Zhengzhou University, Zhengzhou 450000, China; China-US (Henan) Hormel Cancer Institute, Zhengzhou 450000, China; Tianjian Laboratory of Advanced Biomedical Sciences, Zhengzhou University, Zhengzhou 450000, China; The Collaborative Innovation Center of Henan Province for Cancer Chemoprevention, Zhengzhou 450000, China; State Key Laboratory of Esophageal Cancer Prevention and Treatment, Zhengzhou University, Zhengzhou 450000, China; Provincial Cooperative Innovation Center for Cancer Chemoprevention, Zhengzhou University, Zhengzhou 450000, China

**Keywords:** high-throughput omics, ESCC, mechanism, therapeutic target, diagnosis marker, prognosis marker, tumor microenvironment

## Abstract

Esophageal squamous cell carcinoma (ESCC) remains a major health burden, particularly in Asia, with poor patient prognosis despite advancements in radiotherapy, chemotherapy, and immunotherapy. The marked interpatient and intratumor heterogeneity of ESCC underscores the need for molecularly informed diagnostic and therapeutic strategies. Recent high-throughput omics technologies, including genomics, transcriptomics, proteomics, and metabolomics, have substantially advanced our understanding of ESCC biology. Genomic profiling has revealed recurrent alterations such as *TP53* and *NOTCH1* mutations, as well as actionable targets including *PIK3CA*, *FGFR1*, and *SOX2* amplifications, which provide new opportunities for precision therapy. Epigenomic and transcriptomic analyses have identified methylation-based early detection markers (e.g., PAX9, SIM2) and immune-related transcriptomic subtypes associated with prognosis and immunotherapy responsiveness. Proteomic and metabolomic studies have further uncovered cell cycle and spliceosome pathway activation and altered lactate metabolism, offering additional biomarker and therapeutic insights. In this review, we synthesize these multi-omics advances and highlight how they collectively inform improved diagnostic, prognostic, and therapeutic strategies for ESCC. Despite these developments, the clinical translation of multi-omics findings remains limited due to the lack of standardized analytical pipelines, insufficient multicenter validation, and the high cost and technical complexity of integrating multi-omics data into routine clinical workflows. Future research integrating artificial intelligence with multi-omics data holds promise for enhancing diagnostic accuracy and enabling more precise therapeutic decision-making in ESCC.

## Introduction

Esophageal cancer is the eighth most commonly diagnosed malignancy worldwide and the sixth leading cause of cancer-related mortality (Pennathur et al., 2013; Siegel et al., 2016). Survival rates for esophageal cancer remain low, with 5-year survival rates ranging from 10% to 30% in most countries (Enzinger and Mayer, 2003). Esophageal squamous cell carcinoma (ESCC) and esophageal adenocarcinoma (EAC) are the two major histological subtypes of esophageal cancer based on histological features (Morgan et al., 2022). The impact of esophageal cancer varies by country and population, with its prevalence mainly related to underlying risk factors and differences in subtype distribution. While EAC is predominant in Western populations and associated with Barrett’s esophagus, gastroesophageal reflux, and obesity (Pohl et al., 2013; Quante et al., 2018), ESCC accounts for approximately 90% of cases globally and is the predominant subtype in Asia, strongly linked to tobacco use, alcohol consumption, and nutritional factors (Rustgi and El-Serag, 2014). Among these two subtypes, ESCC remains the most urgent clinical challenge due to its high incidence, aggressive behavior, and pronounced inter- and intratumoral heterogeneity. Although surgical resection remains the primary curative option (Li et al., 2021a), patients with locally advanced ESCC often experience recurrence or distant metastasis (Matsui et al., 2022). In addition, the substantial heterogeneity of ESCC complicates early detection, risk stratification, and treatment selection, highlighting the need for deeper molecular interrogation to support precision oncology.

High-throughput omics technologies including genomics, epigenomics, transcriptomics, proteomics, posttranslational modification profiling, and metabolomics enable comprehensive characterization of cancer at multiple biological levels. Whereas single-omics approaches provide insights into key molecules and events at a specific level, integrated multi-omics strategies have the unique capability to reveal regulatory interactions, delineate tumor evolution, and identify actionable pathways across genomic, epigenetic, transcriptional, and proteomic layers. For ESCC, multi-omics studies have substantially shifted the research paradigm from single-gene investigation toward systems-level dissection of tumorigenesis, tumor microenvironment (TME) remodeling, and therapeutic vulnerabilities.

In this review, we summarize recent progress in single-omics and multi-omics research related to ESCC, highlight key molecular alterations and regulatory programs uncovered by these technologies, and evaluate their potential diagnostic, prognostic, and therapeutic implications. We also provide a comparative assessment of different omics strategies and discuss how integrated multi-omics analyses contribute to mechanistic understanding and precision treatment development. Despite the rapid accumulation of omics datasets, significant challenges remain in translating these findings into routine clinical practice, particularly due to the lack of standardized analytical workflows, limited multicenter validation, and complexities associated with integrating multi-dimensional data for clinical decision-making.

## Genomic alterations in ESCC development

**Figure 1.**
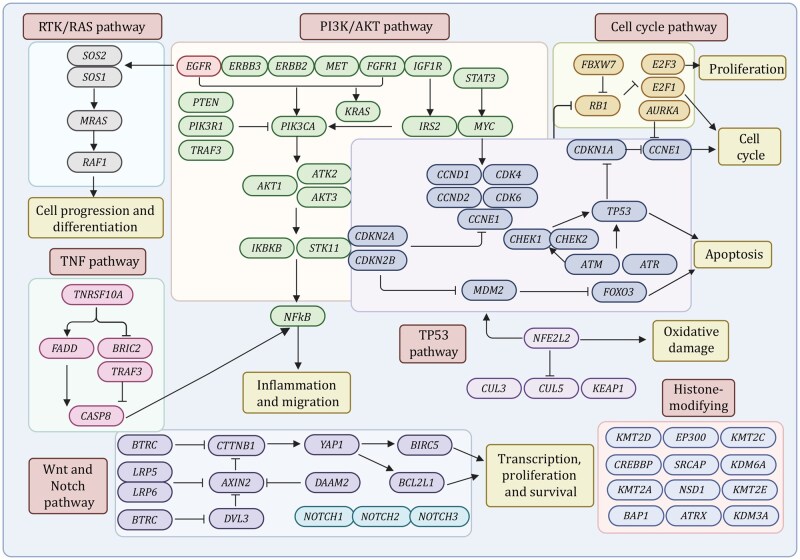
**Genomic landscape and core oncogenic signaling pathways in ESCC**. Genomic studies have demonstrated that ESCC is driven by recurrent alterations in multiple signaling pathways, including RTK/RAS, PI3K/AKT, cell cycle, TP53, TNF, Wnt, and Notch pathways, together with extensive disruption of histone-modifying. Frequent alterations in genes such as *TP53*, *CDKN2A*, *CCND1*, *PIK3CA*, *EGFR*, *NOTCH1*, *NFE2L2*, and epigenetic regulators (*KMT2D*, *KMT2C*, *EP300*, *CREBBP*) collectively promote uncontrolled proliferation, impaired squamous differentiation, cell cycle, inflammatory signaling, and tumor progression.

Advances in next-generation sequencing (NGS) and reductions in sequencing costs have greatly accelerated genomic profiling in ESCC, enabling detailed characterization of single-nucleotide variants (SNVs), copy number alterations (CNAs), and chromosomal rearrangements through whole-exome sequencing (WES) and whole-genome sequencing (WGS). These high-throughput approaches have revealed extensive genomic instability and provided a comprehensive mutational landscape that underpins ESCC initiation, progression, metastatic dissemination, and therapeutic resistance (Fig. 1 and Table 1).

**Table 1.** Representation of genetics studies in ESCC.

Cancer type	Platform	Sample sources	Number of samples	Key mutant genes	Key mutant pathways	Function	References
**ESCC**	WES	Tissue	WES (*n *= 113)	*TP53*, *CCND1*, *CDKN2A*, *NFE2L2*, *RB1*, *KMT2D*, *KMT2C*, *KDM6A*, *EP300*, *CREBBP*, *FAT1*, *FAT2*, *FAT3*, *FAT4*, *AJUBA*, *NOTCH1*, *NOTCH2*, *NOTCH3*, *FBXW7*	Cell cycle, apoptosis, histone modifiers, Hippo and Notch pathways	*EP300* mutations were associated with poor survival	(Gao et al., 2014)
**ESCC**	WGS, WES	Tissue, blood	WGS (*n *= 17), WES (*n *= 71)	*TP53*, *RB1*, *CDKN2A*, *PIK3CA*, *NOTCH1*, *NFE2L2*, *ADAM29*, *FAM135B*, *MIR548K*, *MLL2* (*KMT2D*), *ASH1L*, *MLL3* (*KMT2C*), *SETD1B*, *CREBBP*, *EP300*	Wnt, cell cycle, Notch, RTK-Ras and AKT pathways	*FAM135B* serve as a prognostic marker or therapeutic target	(Song et al., 2014)
**ESCC**	WES	Tissue	WES (*n *= 20)	*TP53*, *PIK3CA*, *NOTCH1*, *FAT1*, *FAT2*, *ZNF750*, *KMT2D*	RTK-MAPK-PI3K, cell cycle and epigenetic regulation pathways	*XPO1* and *FGFR1* serve as therapeutic targets, *ZNF750*, *FAT1* and *FAT2* serve as tumor suppressors	(Lin et al., 2014)
**ESCC**	WGS	Tissue	WGS (*n *= 508)	*TP53*, *FAT1*, *NOTCH1*, *KMT2D*, *CDKN2A*, *FBXW7*, *ZNF750*, *FAT2*, *PIK3CA*, *EP300*, *NFE2L2*, *AJUBA*, *RB1*, *KMT2C*, *CREBBP*, *KMD6A*, *TGFBR2*, *KRT5*, *CDH10*, *LILRB3*, *YEATS2*, *CASP8*	RTK-RAS, MYC, cell cycle, NRF2, NOTCH and WNT pathways	*NFE2L2* serve as tumor suppressor and prognostic marker, *SLC35E2* serve as prognostic marker	(Cui et al., 2020)
**ESCC**	NGS	Tissue	NGS (*n *= 46)	*TP53*, *NOTCH1*, *NFE2L2*, *RB1*, *PTEN*, *CDKN2A*, *PTCH1*, *PIK3CA*	Cell cycle, NOTCH, PIK3‑AKT, MAPK, DNA repair, RTKs signaling pathways	Elucidation the major cancer-related genes	(Munari et al., 2021)
**ESCC**	WES	Tissue	WES (*n *= 28)	*TP53*, *TTN*, *CSMD3*, *SYNE1*, *LRP1B*, *PIK3CA*, *DNAH5*, *NOTCH1*	PIK3CA-AKT pathway	*PIK3CA* serve as potential therapeutic target	(Mangalaparthi et al., 2020)
**ESCC**	WES	Tissue	WES (*n *= 10)	*TP53*, *NCOR1*, *APC*, *KMT2C*, *CDKN1B*, *NOTCH*, *MYC*, *PIK3CA*, *SOX2*, *CCND1*, *SHANK2*, *CTTN*, *KRAS*, *SBS16*, *SBS26*	RTK, homeostasis, EMT, invasion, metastasis, cell cycle, checkpoint, chromatin remodel pathways	*SBS16* increase DNA damage, *SBS26* defects in mismatch repair and microsatellite instability	(Erkizan et al., 2021)
**ESCC**	NGS	Tissue	NGS (*n *= 148)	*CDKN2A*, *FGFR3*, *IKZF1*, *BCL11B*, *NKX2-1*, *SMARCB1*	NA	*CDKN2A* might be important in ESCC development	(Onozato et al., 2021)

**ESCC**	WES, TCR- seq	Tissue, blood	WES (*n *= 185), TCR-seq (*n *= 31)	*TP53*, *NFE2L2*, *BRIP1*, *BRCA1/2*, *NOTCH1*, *ZNF750*, *AXIN2*, *KMT2D*, *EP300*, *KDM6A*, *SMARCA4*, *CREBBP*, *PIK3CA*, *ERBB4*, *MET*, *MTOR*, *FBXW7*, *PTCH1*, *CD274*	Cell cycle, cell differentiation, histone modification, RTK-RAS-PI3K, cell proliferation pathway	*ERBB4* serve as oncogenic gene, *BRCA1/2* serve as therapeutic target	(Yan et al., 2019)
**ESCC**	WGS	Tissue	WGS (*n *= 6)	*H3K9me3*, *H3K27me3*, *EZH2/SUZ12*, *lncRNA ESCCAL-1*	WNT2/β-catenin pathway	DNA methylation gain at the promoter region activates the WNT2/β-catenin pathway	(Cao et al., 2020)
**ESCC**	WGS	Tissue, blood	WGS (*n *= 94)	*CDKN2A*, *TP53*, *NOTCH1*, *FBXW7*, *NFE2L2*, *AJUBA*, *LRP1B*, *TTC28*	RTK/RAS/PI3K, cell cycle, WNT, Notch, TP53 pathway, TNF pathway	*LRP1B* and *TTC28* are the most frequently affected genes by structural variation	(Chang et al., 2017)
**ESCC**	WES	Tissue	WES (*n *= 88)	*TP53*, *NOTCH1*, *PIK3CA*, *NFE2L2*	*RTK/PI3K, NOTCH* family, and *NFE2L2* pathways	Altered gene regulation of imprinted genes and FAT family genes were revealed	(Takemoto et al., 2021)
**ESCC**	WGS	Tissue, blood	WGS (*n *= 4)	*KISS1R*, *AMH*, *MNX1*, *WNK2*, *PRKRIR*, *MACROD2*, *FHIT*, *PARK2*	NA	*MACROD2*, *FHIT* and *PARK2* often intragenic deletions	(Hu et al., 2016)
**ESCC, EAC**	WGS, SNP array	Tissue	WGS (*n *= 51), SNP array (*n *= 113)	*CCND1*, *SOX2*, *TP63*, *ERBB2*, *VEGFA*, *GATA4*, *GATA6*	RTK/RAS/PI3K, cell cycle, cell differentiation, chromatin remodeling	Three molecular subclasses were identified	(Cancer Genome Atlas Research Network et al., 2017)
**ECRT**	WES	Tissue	WGS (*n *= 42)	*TGFBR2*, *IRF2BPL*, *PIK3CA*, *NFE2L2*	DNA-repair, NOTCH family, Hippo pathway, Histone-modifying gene, RTK-RAS pathway, PI3K pathway, cell cycle, Nrf2 pathway	Predict prognosis	(Li et al., 2022)
**ESCC**	WES, SNP array	Tissue	WES (*n *= 144), SNP array (*n *= 144)	*TP53*, *CCND1*, *CDKN2A*, *FBXW7*, *MLL2*, *EP300*, *CREBBP*, *TET2*, *NOTCH1*, *NOTCH3*, *FAT1*, *YAP1*, *AJUBA*, *PIK3CA*, *EGFR*, *ERBB2*, *ADH1B*, *ALDH2*, *CYP2A6*	Cell cycle, epigenetic processes, the NOTCH, WNT and receptor-tyrosine kinase-phosphoinositide 3-kinase signaling pathways	*EP300* and *TET2* correlated with survival times, *ZNF750* associated with the APOBEC signature	(Sawada et al., 2016)

**ESCC**	WES	Tissue	WES (*n *= 302)	*TP53*, *NOTCH1*, *FAT1*, *ZNF750*, *RB1*, *PTCH1*, *PIK3CA*, *FBXW7*, *NFE2L2*, *CDKN2A*, *FAM90A1*, *TNRC6A*, *ZNF750*, *CDC27*	NA	*ZNF750* and *CDC27* are major candidate driver genes	(Guo et al., 2018)
**ESCC**	WGS	Tissue	WGS (*n *= 52)	*CDKN2A*, *FHIT*, *KDM6A*, *RB1*, *FAT*, *NFE2L2*, *ERBB4*, *CUL3*, *PTEN*, *ZNF750*, *NOTCH1*, *MYC*, *PTHLH*	NA	*PTHLH* act as an oncogene	(Cui et al., 2022)
**ESCC**	WES	Tissue	WES (*n *= 186)	*TP53*, *NOTCH3*, *PTPRC*	DNA mismatch repair	Immune microenvironment shapes the tumor evolution and promotes immune evasion of the tumor	(Cui et al., 2023)
**ESCC**	WES	Tissue	WES (*n *= 102)	*TP53*, *MUC16*, *FAT3*, *SYNE1*, *AKAP9*, *FAT4*, *AKAP9*, *MCAF1*, *PGK1*	DNA replication, cell cycle, ECM signaling	*SBS16* associated with alcohol drinking, promoted DNA replication, APOBEC signature was overrepresented in the nonsmoking/nondrinking patients	(Li et al., 2023b)
**ESCC**	WES	Tissue, blood	WES (*n *= 125)	*TP53*, *PIK3CA*, *FAT1*, *ADGB*, *DOPEY1*, *HECW1*, *LAMA1*, *THSD7A*, *USP9Y*, *PRKAG2*, *PLCH2*, *RBPJ*, *NPIPA5*, *MIB2*	TGFβ signaling, WNT signaling	*FAT1*, *ADGB*, *DOPEY1*, *HECW1*, *LAMA1*, *THSD7A*, *USP9Y*, *PRKAG2*, *PLCH2*, *NPIPA5* and *MIB2* serve as potential target, *CCND1* amplification promoted cell proliferation	(Liu et al., 2020b)
**ESCC**	WES	Tissue	WES (*n *= 64)	*PIK3CA*, *NFE2L2*, *MTOR*, *TP53*, *KMT2D*, *ZNF750*, *ERBB4*, *FGFR2*, *BRCA2*, *ATM*	PI3K/MTOR pathway	*ERBB4*, *FGFR2*, *BRCA2*, *ATM*, *TP53* act as candidate actionable targets	(Hao et al., 2016)
**ESCC**	WGS, WES	Tissue	WGS (*n *= 10), WES (*n *= 57)	*VANGL1*, *SHANK2*, *MYBL2*, *FADD*, *MYBL2*	Wnt/β-catenin pathway	*MYBL2* promotes proliferation and metastasis	(Qin et al., 2016)

**ESCC**	WGS, WES	Tissue	WGS (*n *= 14), WES (*n *= 90)	*PIK3CA*, *CBX4*, *CBX8*, *AJUBA*, *ZNF750*, *PTCH1*, *SOX2*	NOTCH signaling, PI3K pathway, p53 pathway, Hedgehog signaling, MAPK pathway, chromatin remodeling, and regulation of cell adhesion	*AJUBA* and *ZNF750* act as tumor suppressors, *SOX2* is the most likely target	(Zhang et al., 2015b)
**ESCC**	WGS	Tissue	WGS (*n *= 155)	*TP53*, *CDKN2A/B*, *NFE2L2*, *CCND1*, *CTTN*, *ANO1*, *ORAOV1*, *SOX2*, *NEUROG1*, *RUNX3*	Cell cycle, NRF2 and SOX2 signaling	Identified potential targets for each subtype	(Liu et al., 2023b)
**ESCC**	WES	Tissue	WES (*n *= 56)	*TP53*, *KMT2D*, *ZNF750*, *IRF5*, *KMT2D*, *TET2*, *KAT2A*	NA	*ZNF750* as a metastasis suppressor, *TP53* missense mutations associated with poor overall survival	(Dai et al., 2017)

NA, not provided by the author.

### Recurrent genomic loci and chromosomal aberrations drive ESCC susceptibility and instability

Genome-wide association studies (GWAS) in high-incidence Chinese populations have identified multiple susceptibility loci linked to ESCC risk. Significant variants include rs7447927 at 5q31.2 (a single-nucleotide polymorphism [SNP] in *TMEM173*), rs1642764 at 17p13.1 (an intronic SNP in *ATP1B2*, near *TP53*), rs35597309 at 6p21.32 (in the HLA class II region) (Wu et al., 2014b), as well as additional risk loci at 5q11, 6p21, 10q23, 12q24, and 21q22 (Wu et al., 2011b). In a subsequent study, nine additional loci were identified, including seven with significant marginal effect on chromosomes 4q23, 16q12.1, 17q21, 22q12, 3q27, 17p13, and 18p11 (Wu et al., 2012). Additionally, variants such as rs1050631 in *SLC39A6* have also been associated with susceptibility and instability (Wu et al., 2013). These findings underscore the role of germline predisposition in shaping population-level ESCC risk.

### Frequently mutated genes act as oncogenic drivers

*TP53* remains the most frequently mutated gene in ESCC (Cancer Genome Atlas Research Network et al., 2017; Testa et al., 2017; Wang et al., 2015). The Cancer Genome Atlas (TCGA) WES analysis further identified recurrent amplification of *CCND1*, *SOX2*, and *TP63*, which activate pathways critical for squamous differentiation such as WNT, Syndecan, and p63 pathways. Across independent cohorts, recurrent mutations have been identified in *TP53*, *NFE2L2*, *MLL2*, *ZNF750*, *NOTCH1*, and *TGFBR*, highlighting the predominance of alterations in squamous lineage regulators and oxidative stress pathways—features that align ESCC genetically with head and neck squamous carcinoma rather than EAC (Cancer Genome Atlas Research Network et al., 2017).

Deep sequencing studies have expanded the mutational landscape of ESCC. Exome sequencing of 113 pairs of tumor and normal tissues confirmed frequent alterations in cell cycle regulators (*TP53, CDKN2A, RB1*), stress-response genes (*NFE2L2*), histone modifiers (*KMT2D, KMT2C, KDM6A, EP300,* and *CREBBP*), and components of the Hippo (*FAT* family) and Notch (*NOTCH* family) pathways (Gao et al., 2014). A subsequent genomic study of 158 ESCC cases confirmed recurrent mutations in major cancer drivers, including *TP53*, *RB1*, *CDKN2A*, *PIK3CA*, *NOTCH1*, and *NFE2L2*, and discovered novel mutations in *ADAM29* and *FAM135B*, along with additional mutations in histone-regulating genes (*KMT2D*, *ASH1L*, *KMT2C*, *SETD1B*, *CREBBP*, and *EP300*) that collectively disrupted WNT, cell cycle, and Notch signaling (Song et al., 2014). Integrated WES and SCNV profiling in larger cohorts further highlighted recurrent mutations in *FAT1*, *FAT2*, *ZNF750*, and *KMT2D*, and together with aberrations in RTK-MAPK-PI3K, cell cycle regulation, and others (Lin et al., 2014). More recently, WGS of 508 ESCC tumor and paired nontumor tissues identified 22 significantly mutated genes, including both previously recognized drivers (e.g., *TP53*, *FAT1*, *NOTCH1*, *KMT2D*, *FBXW7*) and newly implicated genes such as *KRT5*, *CDH10*, *LILRB3*, *YEATS2*, and *CASP8* (Cui et al., 2020). Collectively, these studies reveal a genomic landscape dominated by disruptions in squamous lineage regulators, chromatin remodeling factors, and major oncogenic pathways.

### Geographic and ethnic variation shapes ESCC mutational landscapes

Notable regional differences have been observed in ESCC genomic profiles. For example, mutations in *AJUBA*, *ZNF750*, *FAT1*, and *FBXW7* were enriched in northern but not southern Chinese populations. Brazilian cohorts showed *TP53* and *NOTCH1* as dominant mutations (Munari et al., 2021), whereas Japanese studies reported high frequencies of mutations in *TP53*, *CCND1*, *CDKN2A*, *MLL2*, *NOTCH1/3*, *FAT1*, and *PIK3CA* (Sawada et al., 2016; Urabe et al., 2019b). Indian ESCC samples frequently harbored *TP53*, *CSMD3*, *SYNE1*, *PIK3CA*, and *NOTCH1* mutations (Mangalaparthi et al., 2020), while Iranian cases displayed mutations linked to DNA repair pathways, cell cycle control, and metabolism related to alcohol, folate, and carcinogens (Akbari et al., 2009). African-American patients exhibited *SBS16* and *SBS26* as dominant mutational signatures, along with *MYC*, *PIK3CA*, *SOX2*, *CCTN*, and *KRAS* alterations (Erkizan et al., 2021). These observations demonstrate that ESCC mutational patterns are shaped by genetic ancestry.

### Risk and metastasis-associated mutations

Environmental exposures, particularly alcohol consumption and tobacco use, shape distinct mutational signatures in ESCC. The alcohol-associated E4 signature, characterized by T > C substitutions, correlates with germline variants in alcohol-metabolizing enzymes as well as with alcohol intake (Cui et al., 2020). Consistently, carriers of risk alleles in both *ADH1B* and *ALDH2* who drink alcohol have an approximately fourfold increased ESCC risk compared with noncarriers (Wu et al., 2012), while tobacco chewers exhibited a higher frequency of C: G > A: T transpositions compared with smokers and nontobacco users in India (Mangalaparthi et al., 2020). *CDKN2A* mutations were enriched in nonsmoking, nondrinking female patients, suggesting alternative carcinogenic pathways (Onozato et al., 2021).

Beyond risk factors, specific mutations also drive metastatic progression. Analysis of paired tumors from patients with esophageal and lung squamous carcinomas revealed clonal relatedness in some cases, indicating metastatic spread, whereas others appeared genetically independent (Xue et al., 2020). WES analysis of primary tumors and metastatic lymph nodes identified recurrent mutations in *TP53*, *KMT2D*, *ZNF750*, and *IRF5*—particularly the loss-of-function mutations in *ZNF750*, which may serve as a metastasis suppressor. Mutations in epigenetic regulators (*KMT2D*, *TET2*, *KAT2A*) and deletions of histone variant genes (6p22, 11q23) suggest a shift toward a more plastic, invasive chromatin state during metastasis (Dai et al., 2017).

### Genomic alterations contribute to chemoradio­therapy resistance

Intrinsic and acquired resistance to chemoradiotherapy is strongly linked to specific genomic defects. *MYC* amplification correlates with poor survival and persistent radiotherapy resistance, and *MYC* knockdown resensitizes ESCC cells (Hirata et al., 2021). Additional mechanisms include *SLC7A8* mutations driving multidrug resistance and promoter hypomethylation of genes such as *SLC8A3*, associated with resistance phenotypes and enriched in protein digestion/absorption pathways (Min et al., 2021). These findings support the development of genomically guided patient stratification for combined-modality therapies.

### Genomic distinctions between ESCC and EAC

Although ESCC and EAC are both forms of esophageal cancer, their genomic architectures are highly divergent. ESCC exhibits more frequent CNA amplifications in *CDKN2A*, *EGFR*, *KRAS*, *MYC*, *CDK6*, *MET*, and particularly squamous lineage drivers (*CCND1*, *SOX2*, *TP63*), whereas EAC harbors a different mutational burden (Bandla et al., 2012). *NOTCH1* mutations are present in 21% of ESCC but absent in EAC; notably, their prevalence is higher in North American ESCC compared with Chinese cohorts (Agrawal et al., 2012). WGS studies further show shared alterations in classic cancer genes (*TP53*, *BRCA2*, *ERBB2*, *FBXW7*, *PTCH*, *NF1*) but also identify ESCC-specific loci such as *KISS1R*, *AMH*, *MNX1*, *WNK2*, and *PRKRIR*, as well as recurrent intragenic deletions in *MACROD2*, *FHIT*, and *PARK* (Hu et al., 2016). These distinctions highlight the necessity of subtype-specific diagnostic markers and therapeutic strategies.

## Epigenetic dysregulation contributes to ESCC pathogenesis

Epigenomics investigates genome-wide epigenetic modifications, including DNA methylation, histone modifications, and chromatin remodeling, which regulate gene expression without altering the underlying DNA sequence. Aberrant epigenetic regulation and the resulting transcriptional reprogramming are recognized hallmarks of cancer (Kanwal and Gupta, 2012; Lima et al., 2011; Ma et al., 2016). Although early ESCC studies largely relied on candidate gene approaches, recent multi-omics investigations have provided a more comprehensive view of the ESCC epigenome, revealing dysregulated methylation, enhancer remodeling, and chromatin accessibility patterns that collectively reshape transcriptional programs during tumorigenesis.

### Aberrant DNA methylation leads to gene silencing

DNA methylation remains the most extensively characterized epigenetic alteration in ESCC. A multinational cohort analyzed with the MethylationEPIC array identified tumor-specific methylation changes and highlighted PAX9, SIM2, and THSD4 as diagnostic candidates (Talukdar et al., 2021). Multi-omics profiling further demonstrated that 98% of CpG loci in ESCC are globally hypomethylated, whereas hypermethylation is enriched at polycomb-regulated regions bound by EZH2/SUZ12 (Cao et al., 2020). Additional genome-wide studies reported large numbers of differentially methylated CpG sites and constructed diagnostic or prognostic methylation signatures, including 12-CpG and 4-CpG panels (Xi et al., 2022a), as well as other methylation-based prognostic models (Chen et al., 2020b). Circulating methylation markers such as EPB41L3, GPX3, and COL14A1 offer promise for noninvasive detection (Li et al., 2014c). In a Northeast Indian cohort, genome-wide methylation profiling using the Infinium 450k array identified distinct patterns of hypermethylated and hypomethylated genes, and several corresponding circulating proteins, including IL22RA2, TNFSF13B, SERPINA4, and TAC3, were validated in serum, indicating their potential as noninvasive diagnostic biomarkers (Singh et al., 2015).

Public datasets have expanded methylation profiling efforts, revealing LINE-1 hypomethylation and promoter hypermethylation of classical tumor suppressors including RASSF1, p16INK4a, RUNX3, and APC (Hoshimoto et al., 2015). Integrative expression-methylation analyses (GSE51287 and GSE26886) further identified IL-6, MMP3, MMP9, and SPP1 as potential biomarkers (Peng et al., 2019; Takahashi et al., 2013). TCGA-derived analyses identified methylation-driven prognostic genes such as ABCD1, SLC5A10, and ZNF69 (Lu et al., 2019), and additional survival models have been proposed, including m6A-related signatures (Pu et al., 2022) and population-specific CpG panels (Han et al., 2020; Li et al., 2019b).

### Histone modifications disrupt transcriptional regulation

Although fewer studies have profiled histone marks in ESCC, key regulatory patterns have emerged. Over­expression of EZH2, the H3K27me3 methyltransferase, leads to silencing of tumor suppressor genes and correlates with poor prognosis (Chen et al., 2011). HDAC1 and HDAC2 upregulation promotes proliferative transcriptional programs (Toh et al., 2013). Integrative analyses also reveal enrichment of active histone marks (H3K4me3, H3K27ac) at promoters and enhancers that regulate pathways such as cell cycle progression and EMT, underscoring the contribution of histone regulators to ESCC transcriptional dysregulation (Yi et al., 2020).

### Chromatin remodeling reprograms the ESCC epigenome

Dysregulation of chromatin remodeling complexes, particularly SWI/SNF components such as ARID1A, SMARCA4, and PBRM1, is frequently observed in ESCC and contributes to widespread changes in chromatin accessibility (Nakazato et al., 2016). Loss of ARID1A impairs DNA repair and promotes transcriptional silencing of differentiation-associated genes (Kearns et al., 2025). Integrated ATAC-seq and RNA-seq analyses further reveal ESCC-specific enhancer and super-enhancer activation patterns that drive oncogene expression. Cooperative dysregulation between remodelers and histone modifiers, such as ARID1A loss with EZH2 overactivity, can reinforce stem-like transcriptional programs and promote aggressive tumor phenotypes. These findings highlight chromatin remodeling as a central mechanism in ESCC epigenetic reprogramming and a potential therapeutic vulnerability.

## Transcriptomic alterations in ESCC progression

The transcriptome, unlike the relatively stable genome, reflects the dynamic state of cellular activity across tissues and disease stages. In ESCC, transcriptomic profiling has been widely used to identify dysregulated genes and pathways that drive tumor initiation, progression, and treatment response. Microarray-based approaches and RNA sequencing (RNA-seq) have together generated a large body of data, enabling the discovery of molecular subtypes, prognostic signatures, and potential therapeutic targets.

### Microarray and RNA-seq reveal transcriptional landscapes

**Figure 2.**
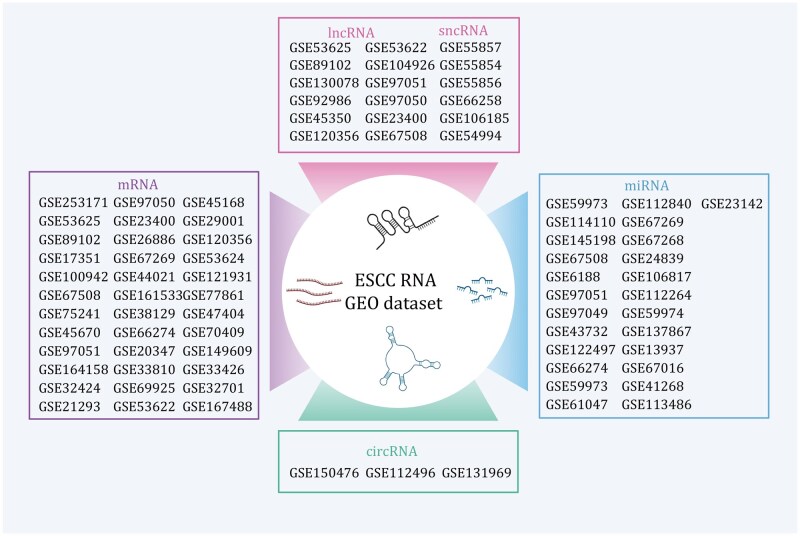
**Public GEO transcriptomic datasets for ESCC**. This figure summarizes Gene Expression Omnibus (GEO) datasets generated from ESCC samples, covering multiple RNA layers, including mRNA, lncRNA, sncRNA, miRNA, and circRNA.

Microarray technology has been extensively applied in ESCC to screen gene expression changes and derive prognostic markers. For example, integration of exome sequencing from 81 tumor–normal pairs with microarray data from 119 patients (GSE53624) identified ANO1 and MMP3 as prognostic genes associated with outcome (Yu et al., 2020). Similarly, RNA-seq analysis of 179 ESCC patients (GSE53625) revealed PLEK2 as a poor prognostic indicator that promotes metastasis and chemoresistance via LCN2 upregulation (Wang et al., 2021a). Other microarray-based analyses (GSE44021, GSE121931, and GSE23400) linked survival to multi-gene expression signatures involving NF1, ASXL1, HSPA4, and additional candidates (Chen et al., 2020b; Su et al., 2011; Yang et al., 2020a). Although many cohorts are relatively small, these datasets, now archived in Gene Expression Omnibus (GEO), together provide a valuable resource for meta-analysis and biomarker validation (Fig. 2 and Table 2).

**Table 2.** Representation of transcriptomics studies in ESCC.

Cancer type	Dataset	Platform	Sample sources	Number of samples	RNA	Key molecular	Function	References
**ESCC**	GSE149609	GPL20301	Tissue	*n *= 10	mRNA	WNT2	WNT2 activate the WNT pathway	(Cao et al., 2020)
**ESC**	NA	Illumina HiSeq2000	Tissue	*n *= 57	mRNA	FAT1, FAT2	FAT1 and FAT2 serve as tumor suppressor genes	(Takemoto et al., 2021)
**ESC**	EGAS00001003832	Illumina HiSeq X Ten	Tissue	*n *= 186	mRNA	HLA-A, HLA-B, B2M	HLA-A, HLA-B, and B2M relate to immune infiltration	(Cui et al., 2023)
**ESCC**	HRA000178	Illumina HiSeq X Ten	Tissue	*n *= 125	mRNA	CCND1	CCND1 associated with poor prognosis	(Liu et al., 2020b)
**ESCC**	HRA003107	Illumina	Tissue	*n *= 155	mRNA	CCND1, CDKN2A, NFE2L2, SOX2, ERBB2	CCND1/CDKN2A related with cell cycle, NFE2L2 related with antioxidant stress response, SOX2 related to transcriptional activation, ERBB2 act as oncogenic signaling	(Liu et al., 2023b)
**ESCC**	GSE44021	GPL571	Tissue	*n *= 113	mRNA	MMP1, SPP1, COL11A1, COL1A1, POSTN, MMP12, MAGEA6, MAGEA3, COL1A2, KRT17, CRISP3, CRNN, MAL, TGM3, CLCA4, SCEL, CRCT1, SLURP1, TMPRSS11E, FLG	Eight genes (NF1, ASXL1, HSPA4, TGOLN2, BAIAP2, EZH2, CHAF1A, SUPT7L) were associated with survival	(Yang et al., 2020a)
**ESCC**	GSE23400	GPL96	Tissue	*n *= 53	mRNA	COL1A2, COL3A1, MET, KRT14, SPINK7/ECG2, HPGD	COL1A1 and COL3A1 related with prognosis	(Su et al., 2011)
**ESCC**	NA	Illumina	Tissue	*n *= 10	mRNA	CLIC3, CLIC4	Low level of CLIC3 and high level of CLIC4 act as prognostic biomarkers	(Liu et al., 2020a)
**ESCC**	PRJNA298963	Illumina HiSeq 2500	Tissue	*n *= 15	mRNA	IL4I1, MAOA, ALDH3A1, ALDH1A3, ALDH3B1, ALDH3B2	Related with tumorigenesis and metastasis	(Cheng et al., 2021)
**ESCC**	PRJCA001577	Illumina X Ten	Tissue	*n *= 119	mRNA	IGH, CD138, MS4A1, IGHV3-74	Related with immunotherapy targeting B cells	(Wang et al., 2022d)
**ESCC**	GSE53624	GPL18109	Tissue	*n *= 119	mRNA	ENST00000435885.1, XLOC_013014, ENST00000547963.1	Three-lncRNA signature act as an independent prognostic factor	(Li et al., 2014a)

**ESCC**	GSE53625	GPL18109	Tissue	*n *= 179	mRNA	ENST00000435885.1, XLOC_013014, ENST00000547963.1	Three-lncRNA signature act as an independent prognostic factor	(Li et al., 2014a)
**ESCC**	GSE53622	GPL18109	Tissue	*n *= 60	mRNA	ENST00000435885.1, XLOC_013014, ENST00000547963.1	Three-lncRNA signature act as an independent prognostic factor	(Li et al., 2014a)
**ESCC**	GSE121931	GPL15207	Tissue	*n *= 125	mRNA	CCND1, CSF3R, E2F2, JUP, RARA and TCF7	Signature can predict the clinical outcome	(Li et al., 2020b)
**ESCC**	GSE20347	GPL571	Tissue	*n *= 17	mRNA	NA	CNNLOH (genomic instability) linked to abnormal gene expression	(Hu et al., 2010)
**ESCC**	GSE17351	GPL570	Tissue	*n *= 5	mRNA	PTGES	PTGES act as a novel target of hypoxia	(Lee et al., 2010)
**ESCC**	GSE38129	GPL571	Tissue	*n *= 30	mRNA	NA	Biallelic loss is significantly associated with reduced mRNA expression	(Hu et al., 2015)
**ESCC**	GSE77861	GPL570	Tissue	*n *= 7	mRNA	NFE2L2	NFE2L2 regulator of detoxification/oxidative-stress defense	(Erkizan et al., 2017)
**ESCC**	GSE47404	GPL6480	Tissue	*n *= 71	mRNA	GRB7	GRB7 act as a potential new therapeutic target	(Sawada et al., 2015)
**ESCC**	GSE29001	GPL571	Tissue	*n *= 12	mRNA	ODC1, POSTN, ASPA	ODC1, POSTN, and ASPA identified as therapeutic targets	(Yan et al., 2012)
**ESCC**	GSE33426	GPL571	Tissue	*n *= 9	mRNA	NA	3D tumor profiling reveals ESCC contains little regional mRNA heterogeneity	(Yan et al., 2013)
**ESCC**	GSE70409	GPL13287	Tissue	*n *= 17	mRNA	MMP1	MMP1 is a potential noninvasive biomarker for detection and prognosis	(Chen et al., 2016)
**ESCC**	GSE75241	GPL5175	Tissue	*n *= 15	mRNA	FOXM1	PIK3R3 is a novel FOXM1 target	(Nicolau-Neto et al., 2018)
**ESCC**	GSE161533	GPL570	Tissue	*n *= 28	mRNA	COL1A1, SPARC, FN1	Associated with ESCC tumor status	(Shen et al., 2021b)
**ESCC**	GSE164158	GPL20795	Tissue	*n *= 8	mRNA	PTTG1	PTTG1 act as a new target	(Chen et al., 2020a)
**ESCC**	GSE26886	GPL570	Tissue	*n *= 10	mRNA	WDR66	WDR66 act as biomarker for risk stratification and drug target	(Wang et al., 2013)
**ESCC**	GSE100942	GPL570	Tissue	*n *= 4	mRNA	RHCG	RHCG act as a transcription regulator	(Ming et al., 2018)
**ESCC**	GSE33810	GPL570	Tissue	*n *= 2	mRNA	CRNN	CRNN act as a tumor suppressor gene	(Chen et al., 2013)

**ESCC**	GSE32424	GPL10999	Tissue	*n *= 7	mRNA	Rab25	Rab25 act as a prognostic biomarker and a novel therapeutic target	(Tong et al., 2012)
**ESCC**	GSE45168	GPL13497	Tissue	*n *= 5	mRNA	NEK6	NEK6 serve as an independent prognostic indicator	(Li et al., 2020a)
**ESCC**	GSE69925	GPL570	Tissue	*n *= 274	mRNA	PRF1, GZMB, IFNG, CASP1, CASP4, TNFSF4, TNFSE11, TNFSF13B, TNFAIP8	Related with immune	(Tanaka et al., 2015)
**ESCC**	GSE45670	GPL570	Tissue	*n *= 28	mRNA	MMP1, LIMCH1, C1orf226	Related with chemoradiotherapy	(Wen et al., 2014)
**ESCC**	GSE32701	GPL570	Tissue	*n *= 20	mRNA	ZEB1, ZEB2, TWIST1	Related with epithelial-mesenchymal transition	(Aoyagi et al., 2011)
**ESCC**	GSE21293	GPL570	Tissue	*n *= 35	mRNA	POSTN	Related with invasion	(Michaylira et al., 2010)
**ESCC**	PRJCA000354	Illumina HiSeq X Ten	Tissue	*n *= 94	mRNA	CUL3, RBPJ	Altered the malignant phenotypes	(Chang et al., 2017)
**ESCC**	NA	Illumina HiSeq2000	Tissue	*n *= 59	mRNA	TP63, KRT5/15, BNC1, DSC3, DSG3	Act as diagnosis markers	(Liu et al., 2016)
**ESCC**	OEP002359	NA	Tissue	*n *= 24	mRNA	FBL	FBL predicts unfavorable patient survival	(Jin et al., 2021)
**ESCC**	GSE167488	GPL24676	Tissue	*n *= 3	mRNA	NA	NA	NA
**ESCC**	TCGA	NA	Tissue	*n *= 95	mRNA	CCND1, SOX2, TP63, ERBB2, VEGFA, GATA4, GATA6	RTK/RAS/PI3K, Cell cycle, Cell differentiation, Chromatin remodeling	(Cancer Genome Atlas Research Network et al., 2017)
**ESCC**	HRA000021	NA	Tissue	*n *= 97	mRNA	ZNF750	Act as a metastatic and prognostic biomarker, and therapeutic target	(Bi et al., 2020)
**ESCC**	GSE253171	GPL30375	Tissue	*n *= 3	mRNA	AMPK	Act as potential therapeutic targets	(Sun et al., 2025)
**ESCC**	GSE106185	GPL570	Tissue	*n *= 23	mRNA	NA	NA	NA

NA, not provided by the author.

RNA-seq has further enabled high-resolution characterization of ESCC transcriptomes. Studies have identified differentially expressed genes, molecular subtypes, metabolic perturbations, immune features, and widespread alternative splicing events. For example, RNA-seq of tumor and matched normal tissues revealed distinct expression patterns among CLIC family members (Liu et al., 2020a), while integration of RNA-seq with targeted metabolomics highlighted dysregulated tyrosine metabolism and associated diagnostic metabolites in primary and metastatic ESCC (Cheng et al., 2021). Immunogenomic analyses using B-cell receptor libraries from 119 tumors and adjacent tissues showed that BCR subtypes correlate with prognosis and immune contexture (Wang et al., 2022d). Subtype classification of 125 tumors identified three ESCC subgroups characterized by differences in metabolic, inflammatory, and proliferative programs, with CCND1 overexpression linked to enhanced proliferation (Liu et al., 2020b). Alternative splicing analyses identified SF3B4 as an overexpressed splicing factor regulating numerous targets and representing a potential therapeutic node (Ding et al., 2021). Additional work has functionally validated transcriptional regulators such as ZNF750 and BRAP as prognostic factors with roles in squamous differentiation, invasion, NF-κB signaling, and lymphangiogenesis (Bi et al., 2020; Zhao et al., 2017). Together, these RNA-based studies delineate a complex transcriptional landscape in ESCC and nominate multiple candidate biomarkers and pathways for clinical translation. Additional RNA-seq-based studies using clinical ESCC samples are summarized in Table 2.

## Dysregulation of ncRNAs in ESCC

ncRNAs, including long noncoding RNAs (lncRNAs), microRNAs (miRNAs), and circular RNAs (circRNAs), are key regulators of gene expression and have emerged as critical players in ESCC biology. High-throughput profiling has revealed that multiple classes of ncRNAs participate in cell cycle control, apoptosis, invasion, metastasis, immune regulation, and therapy response (Table 3).

**Table 3.** Representation of ncRNA profiles in ESCC.

Cancer type	Dataset	Platform	Sample sources	Number of samples	RNA	Key molecular	Function	References
**ESCC**	GSE53625 (GSE53622 and GSE53624)	GPL18109	Tissue	*n *= 358	LncRNA, mRNA	ENST00000435885.1, XLOC_013014, ENST00000547963.1	Three-lncRNA signature act as an independent prognostic factor	(Li et al., 2014a)
**ESCC**	GSE89102	GPL16956	Tissue	*n *= 5	lncRNA, mRNA	linc00460	Act as biomarkers for diagnosis and treatment	(Liang et al., 2017)
**ESCC**	GSE67508	GPL570	Cell	*n *= 2	mRNA, microRNAs	GBP1	GBP1 promotes lymph node metastasis	(Li et al., 2015)
**ESCC**	GSE97051 (GSE97049 and GSE97050)	GPL20115 GPL21572	Tissue	*n *= 7	lncRNA-miRNA-mRNA	lncRNA-TTN-AS1, miR-133b, FSCN1	Act as potential therapeutic targets	(Lin et al., 2018)
**ESCC**	GSE23400	GPL97 GPL96	Tissue	*n *= 5	lncRNA, mRNA	COL1A2, COL3A1, MET, KRT14, SPINK7/ECG2, HPGD	COL1A1 and COL3A1 related with prognosis	(Su et al., 2011)
**ESCC**	GSE67269 (GSE67268 and GSE 44021)	GPL19823	Tissue	*n *= 113	miRNA, mRNA	miR-30e, miR-124, NF1, ASXL1, HSPA4, TGOLN2, BAIAP2, EZH2, CHAF1A, SUPT7L	Associated with survival	(Yang et al., 2020a)
**ESCC**	GSE137867	GPL15207	Tissue	*n *= 4	microRNA, mRNA	hsa-miR-34b-3p, hsa-miR-127-5p, hsa-miR-144-3p, hsa-miR-486-5p, TNF, AKR1C1, AKR1C2, ICAM1, GPR68, GNB4, SERPINE1, MMP12	MMP12 might be a useful tumor biomarker and therapeutic target	(Wang et al., 2021c)
**ESCC**	GSE66274	GPL19823	Tissue	*n *= 30	mRNA miRNA	NA	NA	(Hu et al., 2015)
**ESCC**	GSE120356	GPL23178	Tissue	*n *= 5	lncRNA, mRNA	Lnc-KIAA1244-2	Act as potent therapeutic target	(Ma et al., 2019)
**ESCC**	GSE130078	GPL11154	Tissue	*n *= 23	lncRNA	HERES	Act as an early diagnostic and therapeutic target	(You et al., 2019)
**ESCC**	GSE92986	GPL22868	Tissue	*n *= 141	lncRNA	XLOC_007869, CK327190, XLOC_006476, ASLNC11164, BF894811, BQ376030, RP11-473M20.9	Associated with survival	(Weng et al., 2020)
**ESCC**	GSE45350	GPL13607	Tissue	*n *= 4	lncRNAs, mRNA	ESCCAL_1, HOTAIR	Act as potential diagnostic and prognostic biomarkers	(Cao et al., 2014)

**ESCC**	GSE104926	GPL21290	Tissue	*n *= 6	lncRNA, mRNA	RP5-1092A11.2	Associated with different disease status	(Tian et al., 2020)
**ESCC**	GSE55857 (GSE55854, GSE55856, GSE66258)	GPL10558 GPL14613	Tissue	*n *= 108	sncRNA	miR-223, miR-1269a	Act as prognostic marker	(Jang et al., 2017)
**ESCC**	GSE59973	GPL16770	Tissue	*n *= 3	miRNA	miR-195	Act as a tumor suppressor	(Fu et al., 2013)
**ESCC**	GSE114110	GPL24967	Tissue	*n *= 30	miRNA	miR-424	Act as a novel prognostic marker and an effective therapeutic target	(Wen et al., 2018)
**ESCC**	GSE145198	GPL18044	Tissue	*n *= 4	miRNA	NA	NA	NA
**ESCC**	GSE6188	GPL4508	Tissue	*n *= 31	microRNA	hsa-miR-103/107	Act as diagnostic and prognostic marker	(Guo et al., 2008)
**ESCC**	GSE43732	GPL16543	Tissue	*n *= 119	miRNA	hsa-miR-218-5p, hsa-miR-142-3p, hsa-miR-150-5p, hsa-miR-205-5p	Predict survival	(Chen et al., 2014)
**ESCC**	GSE122497	GPL21263	Serum	*n *= 566	microRNA	miR-8073, miR-6820-5p, miR-6794-5p, miR-3196, miR-744-5p, miR-6799-5p	Act as diagnosis marker	(Sudo et al., 2019)
**ESCC**	GSE112840	GPL23365	Serum	*n *= 52	miRNA	miR-16-5p, miR-451a, miR-574-5p	Act as biomarkers for diagnosis	(Zheng et al., 2018)
**ESCC**	GSE106817	GPL21263	Serum	*n *= 88	miRNA	NA	NA	(Yokoi et al., 2018)
**ESCC**	GSE112264	GPL21263	Serum	*n *= 50	miRNA	NA	NA	(Urabe et al., 2019a)
**ESCC**	GSE59974	GPL19007	Tissue	*n *= 17	miRNA	miR-145-5p, miR-152, miR-193b-3p, miR-376a-3p	Facilitate individualized treatment	(Wen et al., 2016)
**ESCC**	GSE61047	GPL17107	Tissue	*n *= 4	miRNA	microRNA-330-3p	Functions as an oncogene	(Meng et al., 2015)
**ESCC**	GSE13937	GPL8835	Tissue	*n *= 44	miRNA	miR-21, miR-181b, miR-155, miR-146b, miR-375, miR-203	miR-21 associated with prognosis	(Mathé et al., 2009)
**ESCC**	GSE67016	GPL19906	Cell line	*n *= 3	miRNA	miR-214-3p	Act as a tumor suppressor	(Phatak et al., 2016)

**ESCC**	GSE41268	GPL10850	Saliva	*n *= 7	miRNA	miR-10b*, miR-144, miR-451, miR-21	Act as distinctive miRNAs	(Xie et al., 2013)
**ESCC**	GSE113486	GPL21263	Serum	*n *= 40	miRNA	NA	NA	(Usuba et al., 2019)
**ESCC**	GSE23142	GPL7723	Tissue	*n *= 3	miRNA	miR-143, miR-145	Act as anti-oncomirs	(Wu et al., 2011a)
**ESCC**	E-MTAB-2208	NA	Tissue	NA	miRNA	miR-31	miR-31 act as a therapeutic target	(Taccioli et al., 2015)
**ESCC**	GSE150476	GPL21825	Tissue	*n *= 3	circRNA	CircIMMP2L	Act as a biomarker for the diagnosis and prognosis	(Liang et al., 2021)
**ESCC**	GSE112496	GPL21825	Plasma	*n *= 5	circRNA	29 circRNAs, 2 miRNAs, and 10 hub genes	Help understand the circRNA-miRNA-mRNA regulatory mechanism	(Shen et al., 2021a)
**ESCC**	GSE131969	GPL19978	Tissue	*n *= 3	circRNA	circGSK3β	Serve as a novel diagnostic and prognostic biomarker	(Hu et al., 2019)

NA, not provided by the author.

### lncRNAs regulate tumor biology and serve as biomarkers

lncRNAs, defined as transcripts over 200 nucleotides with minimal or no protein-coding capacity (Bazin et al., 2017), are widely dysregulated in ESCC. Numerous studies using GEO and TCGA datasets have constructed lncRNA-based prognostic signatures. Examples include LINC00551 as a candidate prognostic marker (Peng et al., 2021), an eight-lncRNA panel from GSE53625 (Li et al., 2020c), three-lncRNA and seven-lncRNA signatures that predicted overall and disease-free survival (Huang et al., 2018; Mao et al., 2018), as well as additional models derived from GSE92986, GSE53625 and other cohorts (Cao et al., 2022; Li et al., 2014a; Weng et al., 2020; Yu et al., 2019b). These signatures consistently support the prognostic value of lncRNA expression patterns.

Mechanistic studies have shown that individual lncRNAs modulate key oncogenic pathways. LINC00680 acts as a ceRNA for miR-423-5p to regulate PAK6 and promote growth (Xue et al., 2022); HERES and CASC9 interact with EZH2 to modulate Wnt signaling and repress PDCD4, respectively (Wu et al., 2017; You et al., 2019). Other lncRNAs such as Lnc-KIAA1244-2, TN-AS1, and LINC00491 regulate proliferation and apoptosis (Ma et al., 2019; Ye et al., 2021a), while HCG22 suppresses migration via SPINK7/ADAMTS12 (Li et al., 2020d). CASC9 and DUXAP8, identified from multiple public datasets, are associated with metastatic phenotypes (Liu et al., 2018; Pan et al., 2016). Expression profiling in small tissue cohorts further expanded the catalog of cancer-related lncRNAs and mRNAs in ESCC (Wang et al., 2018; Yao et al., 2016).

Recent integrative analyses have linked lncRNAs to immune regulation, autophagy and ferroptosis. An eight-lncRNA immune-related signature was associated with immune checkpoint response (Zhu et al., 2021b); autophagy-related and ferroptosis/iron-metabolism-related lncRNAs were connected to survival and risk stratification (Niu et al., 2022; Zhao et al., 2022d). m^7^G-related lncRNAs and immune-associated multi-lncRNA signatures built from TCGA and GEO datasets were shown to predict prognosis and potential response to immunotherapy (Pang et al., 2021; Zhao et al., 2022a).

lncRNAs also show promise as noninvasive biomarkers. A five-lncRNA panel in plasma exosomes demonstrated diagnostic potential (Jiao et al., 2020), while LINC00324 and LOC100507053 were detected in peripheral blood and proposed as liquid biopsy markers (Sharma et al., 2022). Additional prognostic and functional lncRNAs include LINC01088, which suppresses growth through the NPM1-HDM2-p53 axis (Liang et al., 2023), LINC00022, which promotes tumorigenesis via FTO-mediated m^6^A demethylation (Cui et al., 2021), and LINC01234, which inhibits ESCC progression through miR-193a-5p/CCNE1 regulation (Ma et al., 2020). Network-based analyses have further revealed lncRNA-mRNA regulatory axes and exosome-related lncRNA pairs associated with immune infiltration, microbiota composition, and clinical outcomes (Alaei et al., 2019; Zhao et al., 2022b). Collectively, these findings underscore lncRNAs as versatile regulators with strong potential for biomarker development and therapeutic stratification. A summary of key lncRNA studies is presented in Table 3 and Fig. 2.

### miRNAs act as posttranscriptional regulators in progression

miRNAs are small ncRNAs, typically 20 to 22 nucleotides in length, that fine-tune gene expression posttranscriptionally, and play essential roles in cell proliferation, differentiation, apoptosis, and tumor progression. In ESCC, aberrant miRNA expression has been extensively studied for its diagnostic, prognostic, and therapeutic relevance (Table 3).

Multiple studies have proposed miRNA signatures with strong predictive power. Salivary EV-derived miRNA panels (e.g., miR-1268a, miR-4505, miR-1972, miR-4274, miR-4701-3p, miR-6126) showed potential for early detection (Li et al., 2023a). Expression of hsa-miR-103/107 was associated with poor survival (Guo et al., 2008), whereas miR-30e, miR-124, and miR-574-3p correlated with favorable prognosis or postoperative outcomes (Okumura et al., 2016; Yang et al., 2020a). A four-miRNA model based on GSE43732 predicted survival (Chen et al., 2014), and serum miRNAs such as miR-25, miR-100 and miR-1246 were linked to prognosis and served as circulating biomarkers (Takeshita et al., 2013; Wu et al., 2014a).

Public databases have facilitated the construction of more complex models, including six-miRNA and eight-miRNA signatures derived from GSE43732, TCGA, GSE55856 and other datasets that stratify risk and distinguish early ESCC from healthy controls (Li et al., 2019a; Mao et al., 2019; Miyoshi et al., 2022). Additional panels and serum miRNA sets were associated with disease stage, recurrence, and survival (He et al., 2019; Sun et al., 2018; Wang et al., 2017; Yu et al., 2019c; Zhang et al., 2010). A five-miRNA set (hsa-mir-103-1, hsa-mir-18a, hsa-mir-324, hsa-mir-369, and hsa-mir-320b-2) derived from TCGA showed diagnostic and preventive potential and correlated with survival (Zhao et al., 2015).

Functionally, numerous miRNAs have been implicated in ESCC pathogenesis. Diagnostic serum panels (e.g., miR-8073, miR-6820-5p, miR-6794-5p, miR-3196, miR-744-5p, miR-6799-5p) demonstrated high discriminative power (Sudo et al., 2019), and miRNA-based tests outperformed conventional markers like CEA and CA19-9 in distinguishing ESCC and ESD from healthy controls (Shen et al., 2019). Specific miRNAs such as miR-21-5p, miR-146b-5p, and miR-210-3p were altered in superficial ESCC (Fujihara et al., 2021), while plasma miR-25 and other circulating miRNAs tracked tumor dynamics and diagnosis (Ibuki et al., 2020; Komatsu et al., 2014; Zheng et al., 2018). A four-miRNA model (GSE122497, GSE106817, and GSE112264) demonstrated diagnostic accuracy (Song et al., 2021).

Several miRNAs directly regulate oncogenic pathways and may be exploited therapeutically. miR-130b promotes tumorigenesis via PTEN/Akt signaling (Yu et al., 2015; Zhu et al., 2019a), whereas miR-375 and miR-99a suppress proliferation, metastasis, and IGF1R signaling (Kong et al., 2012; Mei et al., 2017a; Yi et al., 2017). Downregulation of miR-126, miR-143, miR-145, and other tumor-suppressive miRNAs contributes to increased motility and EMT (Liu et al., 2015; Mei et al., 2017b; Wu et al., 2011a; Yang et al., 2019). Additional work has identified apoptosis-related miRNAs (e.g., miR-202 via HSF2 targeting), key regulators from network analyses (miR-181a, miR-202, miR-155), and oncogenic miR-21 driving RASA1 downregulation and EMT (Chen et al., 2019; Meng et al., 2017; Yang et al., 2015). In addition, miR-1 functions as a tumor suppressor (Yao et al., 2015). MicroRNA-384, selected from GSE17351, GSE29001, and GSE45168, suppressed tumor growth by inhibiting LIMK1/cofilin signaling (Yu et al., 2019a).

In addition, a comparative analysis of lncRNA, miRNA, and mRNA expression across normal epithelium, low-grade intraepithelial neoplasia (LGIN), high-grade intraepithelial neoplasia (HGIN), and carcinoma tissues revealed coordinated transcriptomic alterations during ESCC development, particularly during the transition from HGIN to invasive cancer. These findings suggest that different RNA classes participate in shared regulatory programs underlying esophageal tumorigenesis (Li et al., 2014b).

### circRNAs emerge as novel players in ESCC

CircRNAs are covalently closed ncRNAs lacking a 5’ cap or 3’ tails (Kristensen et al., 2019). In ESCC, circRNAs are increasingly recognized for their diagnostic and mechanistic importance. Early circRNA microarray profiling identified multiple dysregulated circRNAs with biomarker potential (Shi et al., 2018). Plasma circRNA profiling revealed hsa_circ_0004771 as a promoter of ESCC progression via the miR-339-5p/CDC25A axis and a candidate noninvasive biomarker. CircCNTNAP3, circCYP24A1, circRUNX1, and circARAP2 have been shown to regulate proliferation, metastasis, and inflammatory signaling through TP53, NF-κB, FOXP3, and FOXM1-related pathways (Gu et al., 2022; Wang et al., 2020a, 2022a; Xu et al., 2022). Conversely, hsa_circ_0001946 exhibits tumor-suppressive properties and predicts recurrence and survival (Fan et al., 2019), while LPAR3 circRNA promotes migration and metastasis via miR-198 sponging (Shi et al., 2020).

Using public datasets, additional functional circRNAs have been identified, including circBCAR3 (hsa_circ_0007624), hsa_circ_0006168, circRNA-DOPEY2, hsa_circ_0000277, and hsa_circ_0006948, which modulate processes such as metastasis and mTOR signaling (Liu et al., 2021b; Pan et al., 2019; Shi et al., 2019; Xi et al., 2022b; Zhou et al., 2021). The circ_0007624/miR-224-5p/CPEB3 axis suppresses tumor progression via EGFR/PI3K/AKT inactivation (Cheng et al., 2022). A four-circRNA signature (hsa_circ_0000005, hsa_circ_0007541, hsa_circ_0008199, and hsa_circ_0077536) has been proposed for risk stratification (Wang et al., 2021e), and circRNA-miRNA-mRNA networks derived from GSE112496 have begun to map the broader posttranscriptional landscape (Shen et al., 2021a).

### RNA modifications alter gene function in ESCC

Emerging evidence has revealed that dysregulated epigenetic modifications, particularly RNA methylation, are critical drivers of tumor initiation, progression, and recurrence (Delaunay and Frye, 2019). Among these modifications, m^5^C is a conserved RNA modification present in rRNA, tRNA, lncRNA, and mRNA (David et al., 2017; Khoddami and Cairns, 2013; Squires et al., 2012). Transcriptome-wide profiling has shown globally increased m^5^C in ESCC, largely driven by overexpression of the methyltransferase NSUN2. Mechanistic experiments demonstrated that NSUN2 stabilizes GRB2 mRNA through an m^5^C-LIN28B-dependent pathway, thereby promoting ESCC initiation and progression (Su et al., 2021). These results highlight the oncogenic role of RNA methylation and suggest that targeting the m^5^C epitranscriptome could represent a novel therapeutic avenue.

## Single-cell transcriptomics uncovers intratumoral heterogeneity

**Figure 3.**
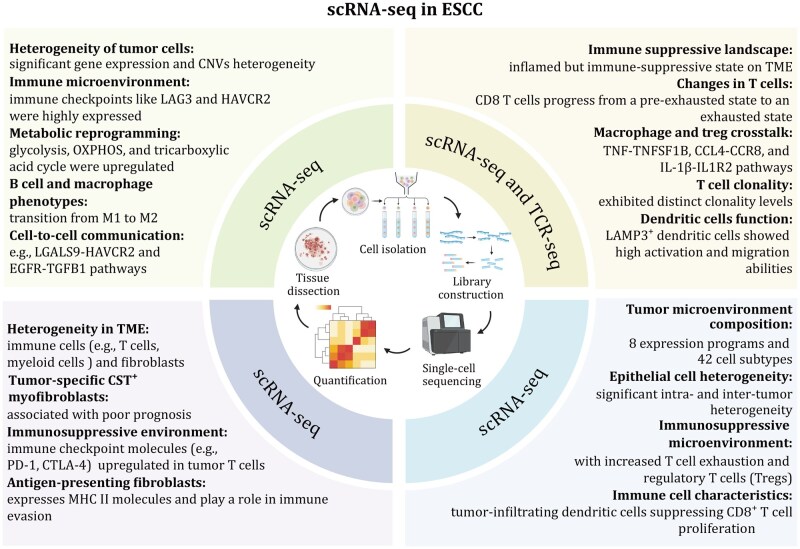
**Overview of scRNA-seq workflow and major findings in ESCC**. This schematic illustrates the general workflow of scRNA-seq, including tissue dissociation, single-cell isolation, library construction, sequencing, and data analysis. It also summarizes the key discoveries from scRNA-seq studies in ESCC, highlighting pronounced tumor cell heterogeneity, metabolic reprogramming (e.g., glycolysis and OXPHOS), and a predominantly immunosuppressive tumor microenvironment. Major findings include T-cell exhaustion with upregulated immune checkpoints, macrophage polarization from M1 to M2, distinct B cell and dendritic cell states, tumor-specific CST1^+^ myofibroblasts associated with poor prognosis, antigen-presenting fibroblasts, and complex cell–cell communication networks shaping ESCC progression.

Single-cell transcriptomics has been extensively studied in ESCC, providing a powerful tool for dissecting tumor heterogeneity and the TME. By analyzing single-cell transcriptomes, researchers can investigate various aspects of the immune landscape of tumors, including immunosuppressive populations, immune cell subsets, and inter- and intratumoral heterogeneity (Fig. 3).

Early scRNA-seq studies analyzing paired ESCC tumors and adjacent nonmalignant tissues have established a comprehensive cellular atlas of the esophageal epithelium and tumor ecosystem. For example, profiling of ∼128,000 cells from five paired samples revealed distinct malignant epithelial states marked by IGFBP2/IGFBP3, ODC1, and SOX4, in contrast to differentiation-associated markers such as CRCT1, CRNN, and SPINK5 in nonmalignant epithelium (Chen et al., 2021c). Importantly, malignant ESCC cells exhibited pronounced metabolic reprogramming, with oxidative phosphorylation contributing substantially to intratumoral metabolic heterogeneity. Transcriptional stratification further identified four malignant cell programs, among which a poorly differentiated state was strongly associated with survival, linking cellular state heterogeneity to clinical outcome. Beyond tumor-intrinsic programs, single-cell analyses consistently demonstrate that ESCC is characterized by a profoundly immunosuppressive TME. Integration of scRNA-seq with TCR sequencing revealed enrichment of exhausted CD8^+^ T cells, regulatory T cells, M2-like tumor-associated macrophages, and tolerogenic LAMP3^+^ dendritic cells, accompanied by depletion of cytotoxic effector populations. Trajectory analyses suggested a transition from pre-exhausted to terminally exhausted T-cell states driven by TOX and immune checkpoint signaling. Several suppressive ligand–receptor interactions, including IL1B-IL1R2 and HLA-I-LILRB1, were identified as potential targets for restoring antitumor immunity, underscoring ESCC-specific immune escape mechanisms (Zheng et al., 2020). Stromal heterogeneity has emerged as another critical determinant of ESCC progression. scRNA-seq studies identified a tumor-enriched CST1^+^ myofibroblast population associated with extracellular matrix remodeling and poor prognosis, as well as an antigen-presenting MHC II^+^ fibroblast subset with potential immunomodulatory roles (Dinh et al., 2021). Expanding these findings, large-scale profiling of over 200,000 single cells from 60 ESCC patients delineated multiple malignant epithelial programs including proliferative, stress-response, antigen-presentation, mucosal-like, and EMT-like states and confirmed a predominantly immunosuppressive microenvironment enriched for exhausted T cells and tolerogenic dendritic cells (Zhang et al., 2021). Notably, a mucosal-like transcriptional program characterized by CXCL17, AGR2, and MUC20 expression correlated with improved survival, highlighting prognostically relevant tumor cell states unique to ESCC.

Recent studies further extend single-cell insights toward therapeutic stratification and biomarker development. Deep immune profiling identified 53 immune cell clusters and demonstrated that PD-L1 blockade can partially reprogram tumor-associated macrophages toward a pro-inflammatory phenotype. Importantly, CD39 (ENTPD1) was shown to more accurately mark tumor-reactive T cells and predict anti-PD-1 response than PD-1 or CD103 alone, suggesting a refined biomarker for immunotherapy selection in ESCC (Chen et al., 2025). In parallel, characterization of the vascular niche revealed a metastatic-enriched GPR116^+^ pericyte subset transcriptionally driven by PRRX1. These pericytes secrete EGFL6, which activates integrin β1-NF-κB signaling in cancer cells to promote invasion and metastasis; targeting this axis suppressed metastasis and enhanced immunotherapy efficacy in preclinical models, while circulating EGFL6 emerged as a potential noninvasive diagnostic and prognostic biomarker (Pei et al., 2025). Collectively, these ESCC-focused single-cell studies move beyond descriptive atlases to reveal disease-defining cellular states, immune escape circuits, and stromal-tumor interactions. While scRNA-seq currently remains a research-intensive platform, the recurrent identification of prognostic tumor programs, immune exhaustion markers, and targetable stromal signaling pathways provides a rational foundation for translating single-cell discoveries into clinically actionable biomarkers and therapeutic strategies for ESCC.

## Proteomic alterations reflect ESCC molecular complexity

Proteomics provides a functional layer of molecular information that complements genomic and transcriptomic findings, offering direct insight into dysregulated pathways and tumor behavior. With advances in mass spectrometry (MS), including quantitative strategies such as stable isotope labeling with amino acids in cell culture (SILAC), isotope-coded affinity tag (ICAT), isobaric tags for relative and absolute quantitation (iTRAQ), tandem mass tags (TMT), as well as label-free methods, researchers are now able to characterize ESCC proteomes with increasing depth and accuracy. These technologies, together with emerging proximity-based protein assays, have facilitated the detection of low-abundance proteins and enabled biomarker discovery from limited clinical material.

Early proteomic studies using two-dimensional difference gel electrophoresis (2D-DIGE) identified proteins involved in structural integrity and cell adhesion, such as a 195-kDa perimembrane protein downregulated in ESCC (Nishimori et al., 2006). TGM3 was subsequently recognized as a prognostic biomarker, with higher expression associated with better survival (Uemura et al., 2009), while elevated PTMA correlated with tumor progression (Zhu et al., 2019b).

Quantitative proteomic approaches have further expanded the ESCC protein landscape. iTRAQ-based analyses identified candidate biomarkers including PSAP, PDIA4, and PLEC1 from paired tumor-normal samples (Pawar et al., 2011), and revealed additional underreported proteins such as proline 4-hydroxylase subunits (Singh et al., 2021). TMT-based profiling uncovered overexpression of ILK, whose inhibition suppressed ESCC proliferation, migration, and invasion, suggesting therapeutic relevance (Ning et al., 2020). Other studies highlighted dysregulated pathways: increased RNA transcription and metabolic activity linked FBL expression to prognosis (Jin et al., 2021), while large-cohort TMT datasets identified thousands of differentially expressed proteins enriched in cell cycle control, DNA repair, immune modulation, EMT, and metabolic reprogramming (Liu et al., 2021a). Proteomic profiling in advanced ESCC further emphasized aberrant cell cycle and spliceosome activity, alongside downregulation of ECM–receptor and focal adhesion pathways (Li et al., 2021c), collectively underscoring profound remodeling of structural and signaling networks during tumor progression. Together, these studies demonstrate that ESCC proteomics not only reveals key tumor-associated pathways but also identifies clinically relevant biomarkers and potential therapeutic targets, strengthening its translational significance.

## Posttranslational modifications influence ESCC biology

**Figure 4.**
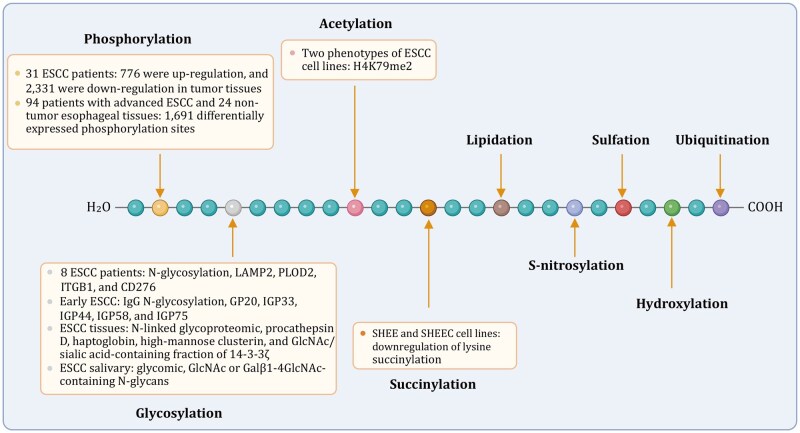
**Representative research progress on PTMs in ESCC**. This figure summarizes the major types of PTMs studied in ESCC, including phosphorylation, acetylation, methylation, ubiquitination, glycosylation, sulfation, lipidation, and hydroxylation. To date, PTM research in ESCC has mainly focused on phosphorylation, glycosylation, acetylation, and succinylation.

Utilizing omics technologies enables the comprehensive detection of a diverse array of posttranslational modifications (PTMs), including phosphorylation, acetylation, succinylation, ubiquitination, glycosylation, sulfation, lipidation, hydroxylation, SUMOylation, nitrosylation, and ADP-ribosylation (Fig. 4). These modifications play pivotal roles in regulating protein function, activating or inhibiting signal transduction pathways, modulating dynamic processes, and controlling gene expression and protein degradation. Common techniques for detecting these modifications include LC-MS/MS combined with specific enrichment methods.

### Phosphoproteomics reveals disrupted signaling pathways

Phosphorylation is the most extensively characterized PTM in ESCC. Large-scale phosphoproteomic analyses have uncovered extensive remodeling of kinase-regulated pathways. A label-free phosphoproteomic study quantified over 67,000 phosphorylation sites and identified thousands of dysregulated phosphosites in ESCC tissues, with enrichment in cell cycle control, EMT, estrogen response path-ways, and hyperphosphorylation of spliceosome-associated proteins (Liu et al., 2021a). Another study profiling advanced-stage ESCC identified 1,691 DEPSs, with upregulated phospho-proteins clustering in cell cycle and spliceosome pathways, whereas phosphorylation levels within ECM-receptor and focal adhesion pathways were reduced (Li et al., 2021c). Together, these findings underscore aberrant phosphorylation signaling as a major driver of ESCC progression.

### Glycoproteomics identifies aberrant glycosylation patterns

Aberrant glycosylation is increasingly recognized as a hallmark of ESCC. Quantitative N-glycoproteomic profiling identified dysregulated glycosylated proteins, including LAMP2, PLOD2, and fucosylated forms of ITGB1 and CD276, which may serve as biomarkers of tumor progression (Gao et al., 2022). Serum IgG glycopatterns further distinguished ESCC, precancerous lesions, and healthy controls, with a composite glycan score showing strong predictive ability and implicating fucosylation and mannose suppression as potential therapeutic targets (Pan et al., 2023). Additional studies identified recurrently altered N-glycoproteins such as procathepsin D and clusterin (Liu et al., 2022b), while salivary glycomic profiling revealed diagnostic N-glycan motifs recognized by DSA lectin (Shu et al., 2021). Database-driven analyses also highlighted GLUT1-associated sialyl-Tn glycoforms as biomarkers for ESCC subtype stratification (Cotton et al., 2021). These results collectively indicate that glycan remodeling plays a functional and clinically relevant role in ESCC.

### Acetylproteomics maps acetylation changes

Lysine acetylation, a key regulator of chromatin structure and transcription, is also altered in ESCC. SILAC-based acetylproteomic profiling in ESCC cell models revealed multiple histone PTMs including acetylation, methylation, and a novel butyrylation event, with H4K79me2 identified as a distinctive modification associated with tumor aggressiveness (Zhang et al., 2015a). These findings highlight acetylation-driven chromatin changes as contributors to ESCC biology.

### Succinylproteomics uncovers novel regulatory mechanisms

Beyond phosphorylation, glycosylation, and acetylation, emerging evidence indicates that additional lysine-based PTMs, particularly succinylation, also contribute to ESCC biology. Succinylation markedly alters lysine residue charge and structure, thereby influencing protein function and cellular metabolism. An integrated multi-omic comparison of immortalized esophageal epithelial cells (SHEE) and their malignantly transformed counterparts (SHEEC) revealed a global reduction in lysine succinylation in ESCC cells (Guo et al., 2021). This widespread loss of succinylation was associated with metabolic reprogramming and enhanced migratory capacity, suggesting that succinylation may act as an unrecognized regulatory node in ESCC pathogenesis and could represent a novel therapeutic vulnerability.

Although several PTMs have now been profiled, many biologically important modifications including lipidation, S-nitrosylation, sulfation, hydroxylation, and ubiquitination remain largely unexplored in ESCC. These PTMs regulate essential processes such as membrane targeting, nitric oxide signaling, protein–protein interactions, collagen stability, and proteasomal degradation. Systematic investigation of these unexplored PTMs will be critical for establishing a more complete ESCC PTM atlas and may reveal additional biomarkers or therapeutic targets.

## Metabolic reprogramming characterizes ESCC

Metabolic reprogramming is a hallmark of ESCC, reflecting the extensive biochemical shifts that sustain malignant transformation, progression, and metastasis. Enabled by analytical platforms such as nuclear magnetic resonance (NMR), MS, ultra-performance liquid chromatography (UPLC), and liquid chromatography-tandem mass spectrometry (LC-MS/MS), metabolomics has uncovered widespread alterations in amino acid, phospholipid, nucleotide, and lipid metabolism in ESCC, offering new opportunities for biomarker discovery and mechanistic insight.

Early plasma- and urine-based metabolomic studies revealed broad disruptions in amino acid pools, phospholipid metabolites, and small-molecule intermediates. Reduced circulating levels of multiple amino acids (Asp, Glu, Gly, His, Thr, Tau, Ala, Met, Ile, Leu, Phe) together with elevated Cys were observed in ESCC patients (Ma et al., 2014), while UPLC-TOF-MS profiling identified increased phospholipid species including phosphatidylserine, phosphatidylcholine, phosphatidylinositol, phosphatidylethanolamine, and sphinganine 1-phosphate—in ESCC plasma (Liu et al., 2013). Urine metabolomics further identified pyroglutamic acid, indole, and p-xanthine as candidate diagnostic metabolites (Xu et al., 2013). Studies comparing metastatic and nonmetastatic ESCC patients showed metabolic shifts involving glycolysis, glutaminolysis, lipid turnover, branched-chain amino acid catabolism, and redox pathways, suggesting metabolic signatures associated with disease aggressiveness (Jin et al., 2014).

Spatial metabolomics has provided additional insight into intratumoral heterogeneity. AFADESI-MSI mapping of tissues from 256 ESCC patients highlighted regional dysregulation in proline biosynthesis, glutamine and uridine metabolism, histidine pathways, fatty acid synthesis, and polyamine metabolism, pointing to PYCR2 and UPase1 as functionally relevant metabolic drivers (Sun et al., 2019). Targeted tryptophan metabolomics also implicated tryptophan–kynurenine axis alterations in both ESCC development and metastasis (Cheng et al., 2017). Lifestyle factors may further shape metabolic risk: smoking was linked to a 3.11-fold increase in ESCC risk, potentially mediated by changes in glutamine, histidine, and cholic acid metabolism (Wei et al., 2021).

Integrated metabolomics-transcriptomics approaches have strengthened biomarker discovery. Dysregulation of glycerophosphatidylcholine metabolism in ESCC tissues led to the identification of PTDSS1 and LPCAT1 as candidate diagnostic genes (Yang et al., 2022), while another tissue-based UPLC/MS study revealed consistent upregulation of amino acid transporters SLC7A5, SLC1A5, and SLC16A10, implicating nutrient transport pathways as potential therapeutic targets (Chen et al., 2021a). Exosome-derived metabolomics further broadened diagnostic potential: profiling of 196 plasma exosome metabolites identified 3′-UMP, palmitoleic acid, palmital, and isobutyl decanoate as predictors of recurrence (Zhu et al., 2021a). A large UPLC-MS/MS study of 450 ESCC patients revealed retinol and linoleic acid metabolism as actionable therapeutic pathways (Wang et al., 2022c). Consistently, plasma GC-MS profiling from patients with ESCC, esophageal squamous dysplasia, and controls demonstrated progressive metabolic changes, including α-tocopherol, cysteine, and aminomalonic acid alterations across disease stages (Yu et al., 2022). Complementary ^1^H-NMR profiling identified creatine and glycine as promising noninvasive liquid biopsy markers (Ouyang et al., 2023).

Together, these studies (summarized in Table 4) demonstrate that ESCC is characterized by profound metabolic rewiring across amino acid metabolism, lipid remodeling, nucleotide pathways, and TME interactions. Metabolomics thus offers both mechanistic insights and a growing repertoire of diagnostic and prognostic biomarkers.

**Table 4.** Representation of metabolomics studies in ESCC.

Cancer type	Platform	Number of samples	Sample sources	Up metabolite	Down metabolite	References
**ESCC**	LC-MS/MS	*n *= 92	Serum	Phenylalanine, 4-hydroxyphenyllactic acid, 3,4-dihydroxyphenylalanine, 3,4-dihydroxyphenylacetic acid and tyrosine	NA	(Cheng et al., 2021)
**ESCC**	HPLC	*n *= 60	Plasma	Cystine	Aspartate, glutamate, glycine, histidine, threonine, taurine, alanine, methionine, isoleucine, leucine, phenylalanine	(Ma et al., 2014)
**ESCC**	UPLC-ESI-TOFMS	*n *= 53	Plasma	Phosphatidylinositol, lithocholyltaurine, phosphatidic acid, L-Urobilinogen, 9'-carboxy-gamatocotrienol, phosphatidyl choline, phosphatidyl ethanolamine, sphinganine 1-phosphate, phosphatidylserine, lysoPC, ganglioside GM2, ganglioside GM2, 12-oxo-20-dihydroxyleukotriene B4	Desmosine/Isodesmosine, 5-β-cyprinol sulfate	(Liu et al., 2013)
**ESCC**	RRLC-MS/MS SRM	*n *= 44	Plasma	L-Carnitine, lactic acid, uric acid, citric acid, linolenic acid, linoleic acid, oleic acid, arachidonic acid	Octanoylcarnitine, nonanoylcarnitine, decanoylcarnitine, undecanoylcarnitine, lysoPC (14:0), lysoPC (16:1), lysoPC (16:0), lysoPC (18:0), lysoPC(20:3), cholic acid	(Xu et al., 2013)
**ESCC**	GC/MS	*n *= 80	Serum	2-Hydroxybutyric acid, hypotaurine, aspartic acid, β-alanine, 3-Hydroxybutyric acid, myristic acid, palmitic acid, oleic acid, linoleic acid, palmitelaidic acid, 1-Monooleoylglycerol, ribose, maltose, lactose, creatinine	Glucose, alanine, lactic acid, glutamine, citric acid, fumaric acid, 1,5-Anhydroglucitol, valine, 2-Ketoisovaleric acid, 2-Ketoisocaproic acid, 3-Methyl-2-oxovaleric acid, tryptophan, indolelactic acid, iminodiacetic acid, phosphoethanolamine, γ-Aminobutyric acid, glycolic acid, cysteine, methylcysteine, α-Tocopherol, γ-Tocopherol, threonine, uric acid, erythritol, inositol, myo-Inositol 1-phosphate, cholesterol, hydroxylamine	(Jin et al., 2014)
**ESCC**	AFADESI-MSI	*n *= 256	Tissue	Arginine and proline metabolism; FA biosynthesis; alanine, aspartate; glutamate metabolism; pyrimidine metabolism; histidine metabolism	NA	(Sun et al., 2019)
**ESCC**	UPLC-MS/MS	*n *= 38	Serum	5-hydroxytryptamine	Tryptophan	(Cheng et al., 2017)
**ESCC**	UHPLC-QTOF/MS	*n *= 464	Serum	Glutamine, histidine, cholic acid	NA	(Wei et al., 2021)

**ESCC**	UHPLC-QTOF/MS	*n *= 60	Tissues	Glycerophospholipid metabolism		(Yang et al., 2022)
**ESCC**	UPLC/MS	*n *= 141	Tissues	Ascorbic acid, formylkynurenine, adenylsuccinic acid, kynurenine, 5-hydroxy-l-tryptophan	Dihydroxyacetone phosphate, ricinoleic acid, 2-hydroxyadipic acid, galactose, and 3-hexaprenyl-4-hydroxybenzoic acid.	(Chen et al., 2021a)
**ESCC**	LC-ESI-MS/ MS	*n *= 91	Plasma	3’-UMP, palmitaldehyde	Palmitoleic acid	(Zhu et al., 2021a)
**ESCC**	GC/MS	*n *= 373	Plasma	Aminomalonic acid	Alpha-tocopherol, cysteine	(Yu et al., 2022)
**ESCC**	LC-MS	*n *= 62	Urine	Pyroglutamic acid, indoxyl, urocanic acid, L-Carnitine, L-Fucose, uric acid, acetylcarnitine, deoxycytidine, phenylacetylglutamine, cAMP, cGMP	Paraxanthine, heptanoylcarnitine, octenoylcarnitine, nonenoylcarnitine, nonanoylcarnitine, decanoylcarnitine, undecenoylcarnitine, undecanoylcarnitine	(Xu et al., 2016)
**ESCC**	^1^H-NMR	*n *= 89	Tissues	NAA, glycine, glutamate, valine, leucine/isoleucine, L-tyrosine, phenylacetylglycine, methionine, phenylalanine, GABA, phenylacetyglutamine, glutamate γ-H, taurine, glycoprotein, acetone, malonate, acetoacetic acid, acetate, trimethylamine, formate, uracil, NAC1, adenine in ATP/ADP and NAD/NADH, NAC2, acetyl hydrazine, hippurate	Creatine, glycine, glutamine, 4-HPPA, phenylacetylglycine, creatinine, L-aspartate, myo-inositol, Cho, choline, glucose, ethanol, α-ketoglutaric acid oxime, AMP, NAD	(Ye et al., 2021b)
**ESCC**	GC/MS LC/MS	*n *= 26	Serum	Glutaric acid, cystine, betaine, glucuronic acid	Xylose, pyruvic acid	(Nishiumi et al., 2019)
**ESCC**	GC/MS, LC/MS	*n *= 26	Serum		Arabitol, betaine, glycine, L-aspartate, L-serine, 3-aminoglutaric acid, L-arginine, uracil, 2-hydroxyglutaric acid	(Fujigaki et al., 2018)
**ESCC**	UPLC-MS/MS	*n *= 50	Urine	Nicotinuric acid, fucose, urocanic acid, phenylacetylglutamine, cAMP, cGMP	Glucuronide	(Xu et al., 2019)
**ESCC**	GC/UPLC-TQ-MS/MS	*n *= 67	Serum	Tryptophan (Trp), L-carnitine, acetyl-carnitine	Citrulline, lysine	(Zhao et al., 2022c)

**ESCC**	GC-TOF/MS	*n *= 34	Sera	Glutamine	Glutamic acid, alanine, glycine, serine, ornithine, ribose, glyceric acid, docosahexaenoic acid, linoleic acid	(Liu et al., 2022a)
**ESCC**	LC-MS/MS	*n *= 40	Serum	Phosphatidylcholines	NA	(Mir et al., 2015)
**ESCC**	GC-TOF/MS	*n *= 46	Plasma	Isocitric acid, linoleic acid, citric acid	L-histidine, 3’4 dihydroxyhydrocinnamic acid	(Zhang et al., 2020b)
**ESCC**	GC-MS	*n *= 74	Serum	L-glutamine	Propanoic acid, linoleic acid, glycerol-3-phosphate	(Zhang et al., 2020a)
**ESCC**	CE-MS and LC-MS	*n *= 28	Tissues	Leucine, isoleucine, valine	NA	(Zang et al., 2021a)
**ESCC**	UHPLC-QTOF/MS	*n *= 653	Serum	Hypoxanthine, inosine, carnitine (14:1), glycochenodeoxycholate, PC (P-18:0/18:3)	Dopamine, L-histidine, 5-hydroxyindoleacetate, L-tryptophan, 2'-O-methylcytidine, PC (14:0/0:0), PC (O-16:1/0:0), PE (18:0/0:0), PC (16:1/0:0), PC (18:2/0:0)	(Li et al., 2021b)
**ESCC**	LC-MS	*n *= 88	Plasma	Arachidonic acid, piperidine, carnitine, 7Z, 10Z, 13Z, 16Z, 19Z-docosapentaenoic acid, sebacic acid, acetoacetic acid	Indoxyl sulfate, PC (14:0/0:0), deoxycholic acid, trimethylamine N-oxide, pipecolic acid, hydrocinnamic acid	(Chen et al., 2021b)
**ESCC**	1H-NMR	*n *= 65	Serum, urine	Leucine, isoleucine, arginine, valine, lysine, glutamate, glutamine, asparagine, choline, phosphocholine and glycine	Lactate, alanine, pyruvate, citrate, creatine, taurine, myo-inositol and glucose	(Liu et al., 2019)
**ESCC**	MALDI-MSI	NA	Tissue	Cholines, amino acids, polyamines, carnitines, nucleosides, organic acids, phosphatidylcholines (PCs), fatty acids (FAs), phosphatidylethanolamines (PEs), phosphatidic acids (PAs), phosphatidylserines (PSs), phosphatidylglycerols (PGs) and phosphatidylinositols (PIs)	NA	(Zang et al., 2021b)
NA, not provided by the author						

## Multi-omics integration elucidates ESCC molecular alterations

The integration of multi-omics data has greatly advanced understanding of ESCC by linking genomic alterations with downstream transcriptional, proteomic, phosphoproteomic, and metabolic changes. Rather than interpreting each omics layer independently, recent studies have demonstrated that combined analyses can uncover convergent pathways, identify actionable vulnerabilities, and refine molecular subtypes with clinical relevance.

One representative study simultaneously profiled the transcriptome, proteome, phosphoproteome, and metabolome in 24 paired ESCC and adjacent tissues, revealing coordinated dysregulation of RNA processing, cell cycle control, and metabolic pathways, and nominating several potential therapeutic targets (Jin et al., 2021). A larger proteogenomic investigation comparing proteomes of 124 paired tissues with phosphoproteomes of 31 paired tissues and previously reported genomic datasets identified alterations in 35 significantly mutated genes. Among them, TP53 protein levels were elevated, whereas ERBB2 and ZNF750 were reduced, supporting their functional roles in ESCC progression. Integrated pathway-level analyses further highlighted aberrant RTK-PI3K, WNT, and Notch signaling, alongside frequent mutations affecting pericyte-associated components (Liu et al., 2021a). At the proteomic and phosphoproteomic level, profiling of 94 primary tumors and 24 nontumor tissues identified CLK1 as a therapeutically relevant node associated with patient prognosis (Li et al., 2021c). Another multi-omics analysis of transcriptome, proteome, and phosphoproteome data from 60 treatment-naïve paired ESCC samples identified TIMMDC1 as a prognostic indicator and CSNK2A1 as a promising drug target in ESCC biology (Zhao et al., 2024).

Large-scale integrative studies have further mapped ESCC carcinogenesis at the systems level. Analysis of 786 tumor samples from 154 patients using genomic, proteomic, and phosphoproteomic profiling revealed six developmental trajectories representing progressive alterations in major cancer pathways; phosphorylation of PGK1 at S203 emerged as a potential therapeutic target (Li et al., 2023b). Similarly, integration of epigenomic, transcriptomic, and proteomic datasets from 155 ESCC cases identified four molecular subtypes, cell cycle activation, NRF2 pathway activation, immunosuppression (IS), and immunomodulation (IM). The IS and IM subtypes exhibited high immune infiltration, and patients classified as IM showed superior responses to immune checkpoint blockade therapies in clinical trials. A 28-feature classifier developed from these datasets predicted IM subtype and anti-PD-1 responsiveness with a sensitivity of 85.7% and specificity of 90% (Liu et al., 2023b).

Collectively, these studies illustrate how multi-omics integration uncovers functionally relevant pathways, identifies biomarkers and therapeutic targets, and refines ESCC molecular taxonomy with translational implications. Representative multi-omics investigations are summarized in Table 5.

**Table 5.** Representation of multi-omics study in esophageal squamous cell carcinoma.

Cancer type	Omic analysis	Platform	Sample sources	No. of samples	Key genes	References
**ESCC**	Genome	WES, TCR repertoire sequencing	Tissue, blood	WES (*n *= 39), TCR repertoire sequencing (*n *= 39)	ERBB, BRCA1/2, CD274	(Yan et al., 2019)
**ESCC**	Genome, Epigenome, Transcriptome	WGS, WGBS, RNA-seq, iTRAQ.	Tissue	WGBS (*n *= 20), WGS (*n *= 6), RNA-seq (*n *= 20), and iTRAQ (*n *= 20)	H3K9me3, H3K27me3, EZH2/SUZ12, lncRNA ESCCAL-1	(Cao et al., 2020)
**ESCC**	Genome	DNA methylation	Tissue	DNA methylation (*n *= 91)	cg23378365, cg06090867 and cg03244277 in the promoters of CYFIP2, UBXN10, AREG, respectively, and cg02370667 in the gene-body of NECAB2	(Xi et al., 2022a)
**ESCC**	Genome, Transcriptome	WGS, RNA-Seq	Tissue, peripheral blood samples	WGS (*n *= 94), RNA-Seq (*n *= 94)	LRP1B, TTC28	(Chang et al., 2017)
**ESCC**	Genome, Transcriptome, epigenome	WES, RNA-Seq, DNA methylation	Tissue	WES (*n *= 88 pairs), RNA-Seq (*n *= 57), DNA methylation (*n *= 67)	NOTCH family genes, RTK/PI3K pathway genes, and NFE2L2 pathway genes	(Takemoto et al., 2021)
**ESCC**	Genome, Transcriptome	WGS, RNA-Seq	Tissue	WGS (*n *= 528), RNA-Seq (*n *= 133),	PTHLH	(Cui et al., 2022)
**ESCC**	Genome, Transcriptome	WES, RNA-Seq	Tissue, blood	WES (*n *= 50), RNA-Seq (*n *= 125)	TP53, PIK3CA	(Liu et al., 2020b)
**ESCC**	Transcriptome, Proteome, Phosphoproteome, Metabolome	RNA-Seq, iTRAQ, GC-MS	Tissue	RNA-Seq (*n *= 24), iTRAQ (*n *= 24), GC-MS (*n *= 24)	FBL	(Jin et al., 2021)
**ESCC**	Proteome, Phosphoproteome	iTRAQ, TMT	Tissue	iTRAQ (*n *= 124), TMT (*n *= 31)	ELOA, SCAF4	(Liu et al., 2021a)
**ESCC**	Proteome, Phosphoproteome	iTRAQ	Tissue	iTRAQ (*n *= 94/24)	CLK1	(Li et al., 2021c)
**ESCC**	Transcriptome, Proteome, Phosphoproteome	RNA-seq, TMT	Tissue	RNA-seq (*n *= 60), TMT (*n *= 6)	TIMMDC1, CSNK2A1	(Zhao et al., 2024)

**ESCC**	Genome, Proteome, Phosphoproteome	WES, label-free	Tissue	WES (*n *= 102), label-free (*n *= 786), label-free (*n *= 145)	TP53, AKAP9, MCAF1, PGK1	(Li et al., 2023b)
**ESCC**	Genome, Epigenome, Transcriptome, Proteome,	WGS, WGBS, RNA-seq, scRNA-seq, proteomics	Tissue	WGS (*n *= 155), WGBS (*n *= 155), RNA-seq (*n *= 155), scRNA-seq (*n *= 155), proteomics (*n *= 73)	CCND1, CDKN2A/B, NFE2L2, KEAP1, CUL3, SOX2, CD56, ERBB2	(Liu et al., 2023b)

## Multi-omics reveals translational potential in ESCC

**Figure 5.**
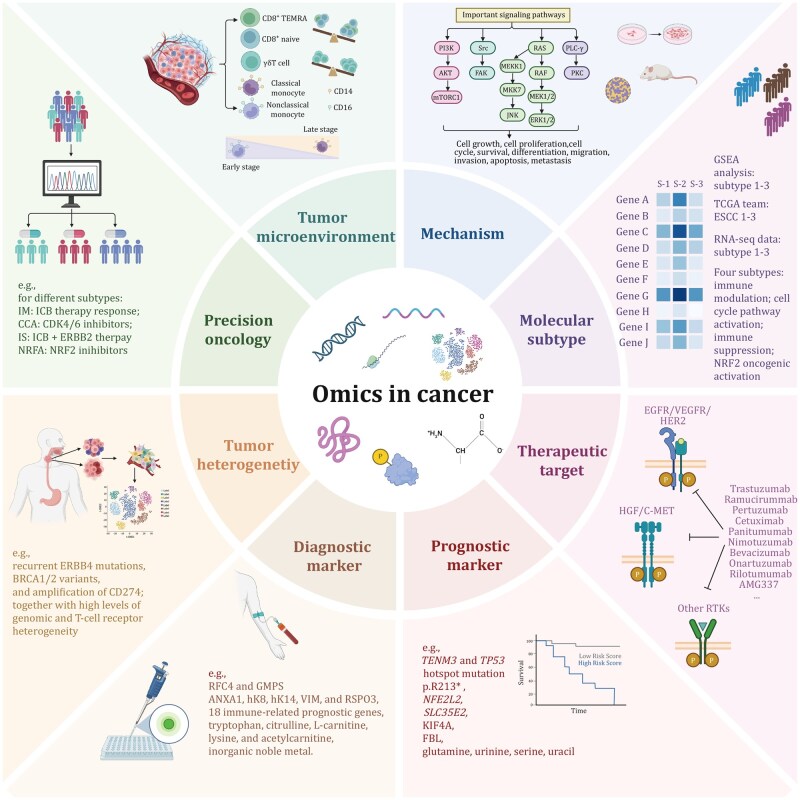
**Multi-omics reveals translational potential in ESCC**. This schematic summarizes how integrative multi-omics analyses (including genomics, transcriptomics, proteomics, metabolomics, and epigenomics) uncover the translational significance of ESCC. Multi-omics approaches elucidate tumor heterogeneity, molecular subtypes, and tumor microenvironmental features; dissect key oncogenic signaling mechanisms; and enable the identification of diagnostic and prognostic biomarkers. In addition, these data support precision oncology by guiding patient stratification and informing the discovery of actionable therapeutic targets, thereby bridging molecular insights with clinical applications in ESCC.

A series of published studies on the omics landscape of ESCC have provided valuable insights for understanding the pathogenesis, molecular subtyping, therapeutic targets, prognostic biomarkers, diagnostic indicators, tumor heterogeneity, TME, and precision treatment of ESCC (Fig. 5). Representative studies have been chosen to exemplify each application.

## Multi-omics uncovers key oncogenic mechanisms

Beyond descriptive cataloging, multi-omics analyses have elucidated mechanistic insights into ESCC initiation and progression. For instance, studies reported that chromosome 3q gains occur during the shift from non-neoplastic tissue to intraepithelial neoplasia, while *TP53* mutations augment DNA replication during this transition (Li et al., 2023b). Alterations such as *AKAP9* and *MCAF1* mutations promote glycolysis and WNT signaling, respectively, at later stages. Regulatory networks have also been uncovered. Wang et al. identified miR-340-5p as a suppressor of PIK3C3, showing that its inhibition promotes proliferation while PIK3C3 re-expression reverses this effect (Wang et al., 2020b). Proteogenomic analysis revealed ribosomal protein FBL as an oncogenic driver that enhances PI3K/AKT activation and accelerates cell cycle progression (Jin et al., 2021). Importantly, Omics approaches can identify dysregulated signaling pathways in ESCC such as PI3K/AKT signaling, Src/FAK signaling, RAS/RAF signaling. Together, these studies demonstrate how multi-omics can resolve stage-specific events and reveal functional convergence on metabolic remodeling, DNA replication stress, and kinase signaling.

## Molecular subtypes of ESCC emerge from integrative clustering

Unsupervised clustering across genomic, transcriptomic, and epigenomic layers has revealed the presence of distinct ESCC molecular subtypes. In Japanese cohorts, two mutational signatures—CpG transitions and APOBEC-mediated substitutions—were linked to environmental exposures (alcohol, smoking) and genetic variants (ALDH2 and CYP2A6) (Sawada et al., 2016). TCGA further classified esophageal cancers into ESCC1, ESCC2, and ESCC3, with clear geographic and molecular distinctions (Cancer Genome Atlas Research Network et al., 2017). Across 1,930 ESCC genomes, an ESCC-meta-set identified 11 mutational signatures and three NMF clusters. Cluster 1, enriched for APOBEC signatures and ZNF750 mutations, exhibited the poorest prognosis and greater metastatic propensity, whereas cluster 3 was characterized by mismatch repair deficiency (Cui et al., 2020). Another study defined three conserved subtypes based on mutational signature activity, with subtype-specific enrichment of CDC27 amplification, ZNF750 mutations, or TP53 dominance (Guo et al., 2018).

Transcriptomic subtyping has similarly identified clinically meaningful groups. GSEA-based classification of 141 tumors defined three subtypes with dominant feature, WNT pathway activation, glycogen-metabolic suppression, and attenuated neutrophil degranulation (Wang et al., 2022b). Additional RNA-seq analyses described metabolic, inflammatory/metastatic, and cell-proliferative subtypes, distinguished by alterations in EMT pathways, integrin/ECM signaling, and MYC-driven cell cycle programs (Liu et al., 2020b). Recent large-scale integrative work further grouped ESCC into four multi-omics subtypes, cell cycle activation, NRF2 oncogenic activation, IS, and IM. Notably, IM tumors displayed high immune infiltration and superior responses to checkpoint blockade in clinical trials, enabling the development of a 28-feature classifier that predicts anti-PD-1 responsiveness with high sensitivity and specificity (Liu et al., 2023b). Additionally, Japanese ESCC cases were clustered into two mutational signatures: an APOBEC-associated signature and an age-related signature (Takemoto et al., 2021). Collectively, these subtyping systems highlight the molecular heterogeneity of ESCC and offer promising frameworks for patient stratification.

## Therapeutic targets are identified via integrative omics

Multi-omics analyses have yielded a range of potential therapeutic targets. Loss of ZNF750 promotes angiogenesis through the DANCR/miR-4707-3p/FOXC2 axis, nominating this pathway as a therapeutic vulnerability in ZNF750-mutant tumors (Bi et al., 2020). CDCA5 activity is enhanced by promoter H3K27 acetylation, promoting proliferation, invasion, and chemoresistance (Xu et al., 2013). PLEK2 drives metastasis and chemotherapy resistance via LCN2 regulation and represents another promising target (Wang et al., 2021a). Multi-omics studies also highlight phosphoglycerate kinase PGK1 (S203 hyperphosphorylated) (Li et al., 2023b) and CLK1 (Li et al., 2021c) as actionable molecules. Additionally, mutated or aberrantly activated genes in ESCC, such as EGFR, VEGFR, HER2, c-MET, and other receptor tyrosine kinases (RTKs), have been explored as therapeutic targets in clinical treatment (Yang et al., 2020b). These findings demonstrate how integrated omics analysis can systematically uncover therapeutic vulnerabilities that may not be apparent from single-layer datasets.

## Multi-omics-based biomarkers for prognosis and diagnosis

Numerous studies have leveraged omics data to identify biomarkers predictive of prognosis, early detection, or treatment response. NFE2L2 mutations correlate with unfavorable prognosis, and recurrent noncoding driver mutations such as SLC35E2 promoter alterations further refine risk stratification (Li et al., 2021c). Overexpression of KIF4A, identified through multi-dataset analysis, also predicts poor clinical outcomes (Wang et al., 2021d). Several metabolites, including glutamine and serine, were associated with survival, revealing a metabolic layer to prognosis (Zhang et al., 2017). A 12-marker diagnostic panel and a four-marker prognostic panel have been proposed for early detection and risk assessment. Alcohol-associated mutational signatures (e.g., *TENM3*, *TP53* p. R213*) serve as independent prognostic markers (Li et al., 2018).

Multi-omics has enhanced biomarker discovery beyond genomics. CNA-driven upregulation of *RFC4* and *GMPS* was linked to early ESCC detection and immune evasion (Wang et al., 2021b). Serum proteins such as ANXA1, hK8, hK14, VIM, and RSPO3 have also emerged as potential diagnostic candidates (Yang et al., 2021). RNA-seq-derived immune signatures identified 18 prognostic immune-related genes (Xiong et al., 2022). Metabolites such as tryptophan, citrulline, and L-carnitine show promise for early diagnosis (Zhao et al., 2022c). Spatial transcriptomics further identified TAGLN2 and CRNN as early-warning markers experimentally shown to promote malignant transformation (Liu et al., 2023a).

## Tumor heterogeneity is resolved by integrative approaches

Tumor heterogeneity encompasses both intratumor heterogeneity and intertumor heterogeneity (Lin and Lin, 2019). Intratumor heterogeneity refers to the biological variations within a single tumor, while intertumor heterogeneity pertains to the phenotypic and molecular differences between tumors in different patients (Gerashchenko et al., 2013; Tabassum and Polyak, 2015; Visvader, 2011; Wolman and Heppner, 1992). Each omics approach reveals the heterogeneity from distinct perspectives.

Multiregion WES and CGH revealed spatially distinct mutations, showing that single-region sampling fails to represent the full genomic landscape (Cao et al., 2020). Multiregion sequencing of 36 ESCC tumors (186 samples) further demonstrated heterogeneity across genomic, epigenomic, and transcriptomic levels driven by persistent chromosomal instability (Cui et al., 2023). Multiregion analysis of tumors by performing WES and TCR sequencing showed region-specific actionable targets, underscoring the clinical need for multiregion evaluation (Yan et al., 2019). Single-cell sequencing adds another dimension by revealing immune cell and stromal heterogeneity, complementing bulk multi-omics approaches.

## Multi-omics reveals microenvironmental interactions

The ESCC TME is shaped by complex interactions between cancer cells, immune populations, and stromal components. Although most immune and stromal cells generally lack recurrent genomic alterations, multi-omics studies show that they undergo profound transcriptomic and proteomic reprogramming driven by tumor-derived signals. Single-cell analyses have revealed immunosuppressive TME features, including T-cell exhaustion, regulatory T-cell accumulation, M2-like macrophage polarization, and tolerogenic dendritic cells, which jointly contribute to immune escape and therapy resistance. For example, one study revealed a reproducible immunosuppressive landscape marked by depletion of CD4^+^/CD8^+^ central memory T cells (TCM) in tumors, while functional assays showed that restoring TCM could enhance antitumor cytotoxicity. On the myeloid side, a PD-L1^+^ tumor-associated macrophage state was linked to clinical benefit, and PD-L1 blockade *ex vivo* reprogrammed TAMs toward a pro-inflammatory phenotype. Notably, CD39^+^ tumor-infiltrating T cells emerged as a TME-associated biomarker correlated with improved prognosis and better response to PD-1 blockade, highlighting its potential utility for immunotherapy stratification in ESCC (Chen et al., 2025). Integrating genomics, transcriptomics, and proteomics has enabled the mapping of ligand–receptor interactions and the identification of candidate immunotherapeutic targets.

## Precision oncology is enabled by multi-omics translation

Multi-omics technologies have greatly accelerated the translation of molecular discoveries into precision oncology for ESCC by integrating genomic, transcriptomic, epigenomic, proteomic, and single-cell-level datasets. Early precision-therapy studies further demonstrate the feasibility of genomics-guided treatment selection in ESCC. Targeted agents such as gefitinib (an EGFR inhibitor) (Kang et al., 2015), dovitinib (an FGFR1 inhibitor) (Guagnano et al., 2012), and PD-L1 inhibitors (Pectasides, 2018) have shown therapeutic potential in selected patients. In addition, recent evidence indicates that CDK4/6 inhibition can enhance the efficacy of EGFR inhibitors in ESCC, suggesting promising combination-treatment strategies (Zhou et al., 2017). Recently, one study stratified ESCC into four distinct molecular subtypes with clear therapeutic implications. The CCA subtype is defined by CCND1 amplification and CDKN2A/B loss, indicating aberrant cell cycle control and suggesting sensitivity to CDK4/6 inhibitors, such as palbociclib. The NRFA subtype is characterized by frequent alterations in NFE2L2, KEAP1, and CUL3, together with SOX2-associated activation of the NRF2 pathway, highlighting oxidative stress adaptation as a dominant feature and proposing NRF2 signaling as a potential therapeutic target. The IS subtype displays substantial immune cell infiltration but with pronounced immunosuppressive characteristics, along with relatively elevated ERBB2 (HER2) protein expression, suggesting that HER2-targeted therapy, possibly in combination with immunotherapy, may be beneficial. In contrast, the IM subtype is enriched for CD8^+^ T cells and macrophages and demonstrates the most favorable clinical response to anti-PD-1 therapy in trial settings (Liu et al., 2023b). Together, these findings illustrate how multi-omics-based stratification enables more accurate patient classification, informs rational therapeutic selection, and provides a practical framework for implementing precision treatment strategies in ESCC.

## Implications of multi-omics in clinical

Among current multi-omics approaches, targeted genomics and DNA methylation profiling represent the most immediately translatable strategies for routine ESCC diagnostics, whereas proteomics, metabolomics, and single-cell approaches currently remain primarily research-oriented. In clinical practice, targeted therapies for ESCC remain limited, but multi-omics approaches have the potential to substantially improve diagnostic and therapeutic strategies by uncovering novel biomarkers, therapeutic targets, and predictors of treatment response (Table 6). Although routine immunohistochemical markers, such as P53, KI67, CK, CK5/6, P40, P63, and CK7, remain widely used, integrated analysis provides more comprehensive guidance for precision medicine, enabling better prediction of individual treatment responses and facilitating personalized therapeutic planning. NGS panels covering more than 500 genes are now widely applied to detect mutations, CNVs, and gene fusions in ESCC specimens, with platforms such as Illumina NovaSeq offering high analytical sensitivity. Actionable alterations frequently observed include ERBB2 (HER2) amplification in 3% to 8% of ESCC, which supports the use of ERBB2 inhibitors; FGFR amplifications or mutations in 8% to 10% of ESCC, suggesting eligibility for FGFR inhibitors; MET amplification (5%–8% of ESCC), predicting potential benefit from MET inhibitors; and EGFR overexpression (30%–50% of ESCC), which may predict response to EGFR inhibitors. PD-L1 and TMB testing are essential for selecting candidates for second-line immunotherapy. Postresistance testing can further reveal bypass activation of EGFR/ERBB pathways, guiding alternative treatment choices.

**Table 6.** Representation of diagnostic marker, therapeutic target, and prognostic marker in ESCC.

Omics	Function	Key molecular
**Genomics**	Diagnostic marker	12-marker diagnostic panel, *RFC4*, *GMPS*
	Therapeutic target	*EGFR*, *BRCA2*, *BRCA1*, *ROS1*, *ALK*, *ERBB2*, *MET*, *BRAF*, *NTRK1*, *FGFR1*, *KRAS*, *RET*, *CD274*, *VEGFA*, *SLC7A8, ZNF750*, *XPO1*, *PIK3CA*, *FAT1*, *ADGB*, *DOPEY1*, *HECW1*, *LAMA1*, *THSD7A*, *USP9Y*, *PRKAG2*, *PLCH2*, *NPIPA5*, *MIB2*, *ERBB4*, *FGFR2*, *ATM*, *TP53*
	Prognostic marker	*EP300*, *TET2*, *FAM135B*, *SLC39A*, 4 promoter/gene-body CpG sites, APOBEC-associated signatures, *NFE2L2*, *TP53* hotspot mutation p. R213*, *SLC35E2*, *TP53*
**Epigenetics**	Diagnostic marker	PAX9, SIM2, THSD4, IL22RA2, TNFSF13B, SERPINA4, TAC3, 12 promoter/gene body DNA methylation CpG sites, EPB41L3, GPX3, COL14A1, IL-6, MMP3, MMP9, SPP1
	Therapeutic target	HDAC, HMT, HDM, HAT, LSD1
	Prognostic marker	ABCD1, SLC5A10, SPIN3, ZNF69, ZNF608, YTHDF3, RBM15, KIAA1429, ALKBH5
**Transcriptomics**	Diagnostic marker	TP63, KRT5/15, BNC1, DSC3, DSG3, HERES, Linc00460, five plasma exosome lncRNAs (NR_039819, NR_036133, NR_003353, ENST00000442416.1, and ENST00000416100.1), miR-1246, serum miRNA discriminant model (miR-8073, miR-6820-5p, miR-6794-5p, miR-3196, miR-744-5p, and miR-6799-5p), miR-16-5p, miR-451a, miR-574-5p, miR-196a-5p, miR-1-3p, 4-miRNA signature, 7 serum miRNAs (miR-10a, miR-22, miR-100, miR-148b, miR-223, miR-133a, and miR-127-3p), Five miRNAs (hsa-mir-103-1, hsa-mir-18a, hsa-mir-324, hsa-mir-369, and hsa-mir-320b-2), ESCCAL_1, HOTAIR, miR-424, hsa-miR-103/107, circIMMP2L, circGSK3β, EGFL6
	Therapeutic target	PTGES, GRB7, ODC1, POSTN, ASPA, CCND1, SF3B4, miR-130b, NSUN2, PTTG1, WDR66, Rab25, ZNF750, AMPK, Lnc-KIAA1244-2, m7G-lncRNAs, lncRNA-TTN-AS1, miR-133b, FSCN1, MMP12, Lnc-KIAA1244-2, HERES, miR-424, miR-145-5p, miR-152, miR-193b-3p, and miR-376a-3p, miR-31, IL1B-IL1R2, HLA-I-LILRB1
	Prognostic marker	ANO1, MMP3, PLEK2, ZNF750, BRAP, CCND1, NF1, ASXL1, HSPA4, TGOLN2, BAIAP2, EZH2, CHAF1A, SUPT7L, COL1A1, COL3A1, CLIC3, CLIC4, MMP1, Rab25, NEK6, FBL, ZNF750, Linc00460, COL1A1, COL3A1, eleven-gene combination, BRAP, LINC00551, miR-30e, miR-124, NF1、ASXL1、HSPA4、TGOLN2、BAIAP2、EZH2、CHAF1A, SUPT7L, eight-lncRNA signature (including AP000487, AC011997, LINC01592, LINC01497, LINC01711, FENDRR, AC087045, AC137770), seven-lncRNA signature, six-lncRNA signature (AP000696.2, LINC01711, RP11-70C1.3, AP000487.5, AC011997.1, and RP11-225N10.1), m7G-lncRNAs, lncRNAs (FIRLs), three-lncRNA signature (ENST00000435885.1, XLOC_013014 and ENST00000547963.1), miRNA signature (higher levels of EVP miR-1268a and miR-4505 and lower levels of EVP miR-1972, miR-4274, miR-4701-3p, and miR-6126), four-miRNA signature, miR-1246, six-miRNA signature (has-mir-142-3p, has-mir-148b, has-mir-130b, has-mir-365a, has-mir-370, and has-mir-423), five-miRNA signature (miR-181c-5p, miR-195-5p, miR-203, miR-212-3p, and miR-28-5p), ZBTB16, AQP4, ADCYAP1R1, VIPR2, miR-182-5p, miR-455-5p, hsa_circ_0004771, KIF4A, 4-marker prognostic panel, 18 immune-related Genes, XLOC_007869, CK327190, XLOC_006476, ASLNC11164, BF894811, BQ376030, RP11-473M20.9, ESCCAL_1, HOTAIR, miR-223, miR-1269a, hsa-miR-103/107, hsa-miR-218-5p, hsa-miR-142-3p, hsa-miR-150-5p, hsa-miR-205-5p, circIMMP2L, circGSK3β, CXCL17, AGR2, MUC20, EGFL6
**Proteomics**	Diagnostic marker	ANXA1, hK8, hK14, VIM, RSPO3

	Therapeutic target	CSNK2A1, CLK1, PGK1, ILK
	Prognostic marker	TGM3, ILK, FBL, CLK1, TIMMDC1
**PTMs**	Diagnostic marker	GlcNAc or Galβ1-4GlcNAc-containing N-glycans
	Therapeutic target	Fucosylation, mannose, PGK1 S203
**Metabolomics**	Diagnostic marker	Phe, 4-hydroxyphenyllactic acid, 3,4-dihydroxyphe, 3,4-dihydroxyphenylacetic acid, tyrosine, PTDSS1, LPCAT1, tryptophan, citrulline, L-carnitine, lysine, acetylcarnitine
	Therapeutic target	SLC7A5/LAT1, SLC1A5/ASCT2, SLC16A10/MCT10

Several genomic alterations identified through multi-omics studies have demonstrated tangible clinical relevance. Mutations in TP53, EGFR, and ERBB2, common in both ESCC and EAC, are now incorporated into predictive clinical decision-making. In practice, EGFR amplification supports the use of EGFR tyrosine kinase inhibitors such as gefitinib, whereas ERBB2 overexpression serves as a biomarker for trastuzumab treatment. These biomarkers can be assessed using standardized NGS assays that have become increasingly feasible for routine laboratories due to reductions in cost and turnaround time. Commercial diagnostic panels already include multiple ESCC-related genes, allowing integration of genomic testing into clinical workflows.

## Future perspectives and discussion

**Figure 6.**
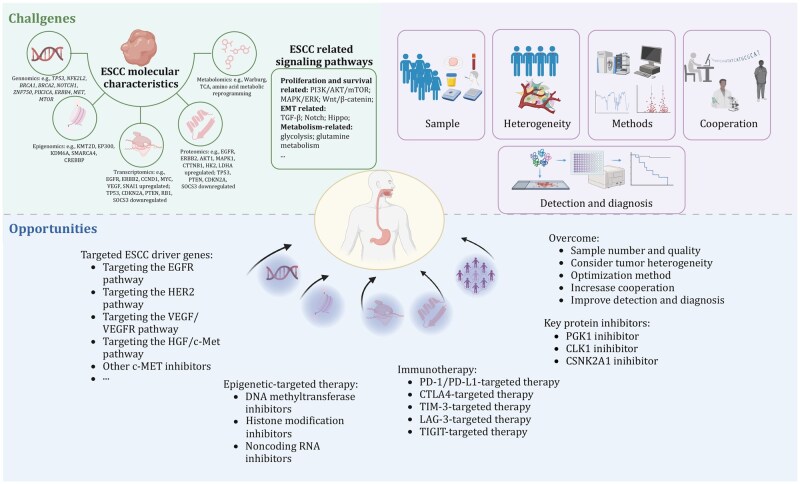
**Opportunities and challenges of multi-omics studies in ESCC**. This schematic summarizes the major challenges and opportunities associated with applying multi-omics technologies to ESCC. Multi-omics profiling reveals key molecular characteristics of ESCC across genomic, epigenomic, transcriptomic, proteomic, and metabolomic layers, and highlights dysregulated oncogenic signaling pathways involved in proliferation, metabolism, epithelial–mesenchymal transition, and immune regulation. However, clinical translation is limited by challenges including sample availability and quality, intratumoral heterogeneity, methodological variability, insufficient multicenter cooperation, and limitations in detection and diagnosis. On the opportunity side, multi-omics data enable the identification of actionable driver genes and pathways (e.g., EGFR, HER2, VEGF/VEGFR, HGF/c-MET), epigenetic targets, immune checkpoints, and key metabolic or signaling proteins, providing a foundation for targeted therapy, immunotherapy, and epigenetic-based treatment strategies.

Although multi-omics studies have greatly advanced our understanding of ESCC, revealing TP53-driven genomic instability, widespread hypomethylation, enhancer remodeling, metabolic reprogramming, and a highly immunosuppressive TME, most discoveries rely on high-cost platforms such as WGS, scRNA-seq, and spatial transcriptomics. These approaches, while mechanistically transformative, are not readily adoptable by routine service laboratories, which often lack standardized workflows, bioinformatic capacity, and access to specialized instrumentation. Consequently, a major gap persists between research-grade molecular insights and clinically deployable tools for ESCC (Fig. 6).

From the perspective of translational utility, genomics and DNA methylation profiling currently offer the clearest path to clinical implementation. Recurrent ESCC alterations such as TP53 and NOTCH1 mutations, CDKN2A deletions, and robust CpG methylation signatures can be adapted to targeted NGS or PCR-based assays compatible with existing clinical workflows. By contrast, proteomic and metabolomic biomarkers (e.g., PGK1 phosphorylation, tryptophan-pathway metabolites) exhibit platform-dependent variability and require MS expertise, limiting routine clinical adoption. Likewise, single-cell and spatial omics provide unparalleled mechanistic resolution (e.g., CST1^+^ CAFs, exhausted T-cell states) but currently function more as discovery tools than practical diagnostics. In addition, the lack of standardized analytical pipelines (e.g., for sample processing, tumor heterogeneity assessment, and data analysis), insufficient multicenter validation and collaboration, limitations in detection and diagnostic frameworks, and the high cost and technical complexity of integrating multi-omics data into routine clinical workflows remain major challenges.

To facilitate broader clinical translation in ESCC, future efforts should prioritize converting high-dimensional multi-omics discoveries into affordable, robust, and standardized assays. In the near term, realistic candidates for routine laboratory implementation include ESCC-specific DNA methylation panels, targeted sequencing panels covering actionable ESCC drivers (e.g., *EGFR*, *HER2*, *VEGF*/*VEGFR*, *HGF*/*c-MET*) and epigenetic regulators (e.g., DNA methyltransferases and histone-modification genes), as well as immune biomarkers (e.g., PD-1/PD-L1, CTLA4, TIM-3, LAG3, TIGIT) and selected protein markers (e.g., PGK1, CLK1, CSNK2A1). The development of automated, ESCC-tailored bioinformatics pipelines will further lower implementation barriers, especially for laboratories without dedicated computational expertise. Meanwhile, rigorous assessment of clinical utility remains essential: candidate metabolomic markers (e.g., creatine, α-tocopherol) and proposed therapeutic targets (e.g., PGK1, CLK1, CSNK2A1) require multicenter validation before adoption. Finally, multi-omics-defined ESCC subtypes, such as immune-modulated and NRF2-activated states, should be tested in prospective clinical trials to determine their value for treatment stratification, and future work integrating artificial intelligence with multi-omics data may further improve diagnostic accuracy and enable more precise therapeutic decision-making in ESCC.

## Conclusions

Multi-omics investigations have substantially reshaped the understanding of ESCC by delineating its genomic instability, epigenetic deregulation, metabolic rewiring, and immune microenvironment heterogeneity, thereby revealing new biomarkers and therapeutic vulnerabilities. Yet, the translation of these insights into routine clinical practice remains limited, as many discoveries rely on resource-intensive platforms and lack cross-cohort validation. Future efforts should prioritize distilling complex multi-omics signals into robust, cost-effective assays compatible with standard diagnostic laboratories, supported by harmonized analytical pipelines and multicenter ESCC cohorts. Integrating genomic, methylation, and immune signatures into prospective clinical trials will be essential for defining molecular subtypes with predictive value. With continued refinement, multi-omics-driven approaches hold strong potential to advance early detection, personalized risk stratification, and the development of targeted therapies, ultimately improving clinical management and patient outcomes in ESCC.

## Abbreviations

ABCD1, ATP binding cassette subfamily D member 1; ADAM29, ADAM metallopeptidase domain 29; ADAMTS12, ADAM metallopeptidase with thrombospondin type 1 motif 12; ADGB, androglobin; ADH1B, alcohol dehydrogenase 1B (class I), beta polypeptide; AGR2, anterior gradient protein 2 homolog; AJUBA, ajuba LIM protein; AKAP9, A-kinase anchoring protein 9; AKR1C1, aldo-keto reductase family 1 member C1; AKR1C2, aldo-keto reductase family 1 member C2; ALDH1A3, aldehyde dehydrogenase 1 family member A3; ALDH2, aldehyde dehydrogenase 2 family member; ALDH3A1, aldehyde dehydrogenase 3 family member A1; ALDH3B1, aldehyde dehydrogenase 3 family member B1; ALDH3B2, aldehyde dehydrogenase 3 family member B2; AMH, anti-Mullerian hormone; AMPK, protein kinase AMP-activated catalytic subunit alpha 1; ANO1, anoctamin 1; ANXA1, annexin A1; APC, APC regulator of WNT signaling pathway; ARAP2, ArfGAP with RhoGAP domain, ankyrin repeat and PH domain 2; AREG, amphiregulin; ARID1A, AT-rich interaction domain 1A; ASH1L, ASH1 like histone lysine methyltransferase; ASPA, aspartoacylase; ASXL1, ASXL transcriptional regulator 1; ATM, ATM serine/threonine kinase; ATP1B2, ATPase Na+/K+ transporting subunit beta 2; AXIN2, axin 2; B2M, beta-2-microglobulin; BAIAP2, BAR/IMD domain containing adaptor protein 2; BCL11B, BCL11 transcription factor B; BLM, RecQ-like DNA helicase BLM; BNC1, basonuclin zinc finger protein 1; BRAP, BRCA1 associated protein; BRCA1, BRCA1 DNA repair associated; BRCA2, BRCA2 DNA repair associated; BRIP1, BRCA1 interacting DNA helicase 1; C1orf226, chromosome 1 open reading frame 226; CASC9, cancer susceptibility 9; CASP1, caspase 1; CASP4, caspase 4; CASP8, caspase 8; CBX4, chromobox 4; CBX8, chromobox 8; CCND1, cyclin D1; CCNE1, cyclin E1; CD138, syndecan 1; CD274, CD274 molecule; CD276, CD276 molecule; CDC25A, cell division cycle 25A; CDC27, cell division cycle 27; CDCA5, cell division cycle associated 5; CDH1, cadherin 1; CDH10, cadherin 10; CDK4, cyclin-dependent kinase 4; CDK6, cyclin dependent kinase 6; CDKN1B, cyclin dependent kinase inhibitor 1B; CDKN2A, cyclin dependent kinase inhibitor 2A; CDKN2B, cyclin-dependent kinase 4 inhibitor B; CHAF1A, chromatin assembly factor 1 subunit A; CLCA4, chloride channel accessory 4; CLIC, chloride intracellular channel; CLIC3, chloride intracellular channel 3; CLIC4, chloride intracellular channel 4; CLK1, CDC like kinase 1; CNTNAP3, contactin associated protein family member 3; COL11A1, collagen type XI alpha 1 chain; COL14A1, collagen type XIV alpha 1 chain; COL1A1, collagen type I alpha 1 chain; COL1A2, collagen type I alpha 2 chain; COL3A1, collagen type III alpha 1 chain; CRCT1, cysteine rich C-terminal 1; CREBBP, CREB binding protein; CRISP3, cysteine rich secretory protein 3; CRNN, cornulin; CSF3R, colony stimulating factor 3; CSMD3, CUB and Sushi multiple domains 3; CSNK2A1, casein kinase 2 alpha 1; CTLA4, cytotoxic T-lymphocyte protein 4; CTTN, cortactin; CUL3, cullin 3; CXCL17, C-X-C motif chemokine 17; CYFIP2, cytoplasmic FMR1-interacting protein 2; CYP24A1, cytochrome P450 family 24 subfamily A member 1; CYP2A6, cytochrome P450 family 2 subfamily A member 6; DANCR, putative uncharacterized protein DANCR; DNAH5, dynein axonemal heavy chain 5; DOPEY1, protein DOP1A; DOPEY2, DOP1 leucine zipper like protein B; DSC3, desmocollin 3; DSG3, desmoglein 3; DUXAP8, double homeobox A pseudogene 8; E2F2, E2F transcription factor 2; ECG2, serine peptidase inhibitor Kazal type 7; EGFL6, epidermal growth factor-like protein 6; EGFR, epidermal growth factor receptor; ELOA, elongin-A; EP300, E1A binding protein p300; EPB41L3, erythrocyte membrane protein band 4.1 like 3; ERBB2, erb-b2 receptor tyrosine kinase 2; ERBB4, erb-b2 receptor tyrosine kinase 4EZH2, enhancer of zeste 2 polycomb repressive complex 2 subunit; FADD, Fas associated via death domain; FAK, focal adhesion kinase; FAM135B, protein FAM135B; FAM90A1, family with sequence similarity 90 member A1; FAT, protocadherin Fat; FAT1, FAT atypical cadherin 1; FAT2, FAT atypical cadherin 2; FAT3, FAT atypical cadherin 3; FAT4, FAT atypical cadherin 4; FBL, fibrillarin; FBXW7, F-box and WD repeat domain containing 7; FGFR, fibroblast growth factor receptor; FGFR1, fibroblast growth factor receptor 1; FGFR2, fibroblast growth factor receptor 2; FGFR3, fibroblast growth factor receptor 3; FHIT, fragile histidine triad diadenosine triphosphatase; FLG, filaggrin; FN1, fibronectin 1; FOXC2, forkhead box protein C2; FOXM1, forkhead box M1; FOXP3, forkhead box P3; FSCN1, fascin actin-bundling protein 1; FTO, FTO alpha-ketoglutarate dependent dioxygenase; GATA4, GATA binding protein 4; GATA6, GATA binding protein 6; GBP1, guanylate binding protein 1; GLUT1, solute carrier family 2, facilitated glucose transporter member 1; GMPS, guanosine monophosphate synthase; GNB4, G protein subunit beta 4; GPR116, adhesion G protein-coupled receptor F5; GPR68, G protein-coupled receptor 68; GPX3, Glutathione p
